# Evaluating Linker Architecture in RNA-Detecting Riboglow Probes and Effects on Fluorescence Turn-On

**DOI:** 10.1021/acschembio.5c00917

**Published:** 2026-02-02

**Authors:** Luke K. Shafik, Gareth M. Francis, Giulia Chitu, Jenna Hanson, Sebastian Lis, Kiera Cunningham, Brooke Tatarian, Aaron R. Van Dyke, Esther Braselmann

**Affiliations:** † 8368 Georgetown University, 3700 O St. NW, Washington, District of Columbia 20057, United States; ‡ 3303 Fairfield University, 1073 N. Benson Rd, Fairfield, Connecticut 06824, United States

## Abstract

Riboglow probes are small molecules where a synthetic fluorophore is connected to an RNA-binding moiety via a chemical linker. Upon binding a short RNA sequence, probe fluorescence intensity and lifetime increase. The fluorescence change is modulated by the architecture of the chemical linker. Here, we systematically interrogated the linker composition in a series of Riboglow probes and assessed fluorescence properties. We found that glycine linkers result in higher fluorescence turn-on compared to a polyethylene glycol linker of similar length. When varying the length of the polyglycine linker, we found that increasing the number of glycine residues led to more substantial fluorescence turn-on upon RNA-ligand binding. Surprisingly, the composition of the Riboglow chemical linker influences fluorescence lifetime contrast when comparing probe binding to two different RNA ligands, a quality necessary for RNA multiplexing. Finally, evaluating probe fluorescence lifetimes in live mammalian cells demonstrated the ability of new Riboglow probes to visualize RNAs live. Insights gained from the systematic assessment of the linker’s architecture will dictate the rational design of future fluorophore-quencher probe designs.

## Graphical abstract

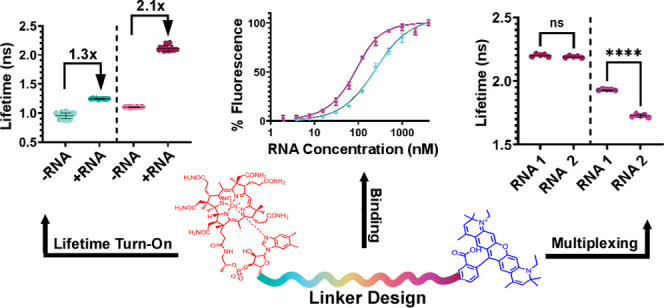

## Introduction

Our understanding of the role RNAs play in cellular function has greatly complexified,^1,2^ necessitating fluorescent markers to genetically tag and track RNAs in live cells, analogous to fluorescent proteins (FPs).^3,4^ The lack of naturally occurring fluorescent RNAs requires the design and subsequent optimization of fluorescent RNA tags that can be genetically fused to an RNA of interest as biosensors. Early generations of fluorescent RNA tags consist of short RNAs that bind proteins fused to FPs, like the bacteriophage-originating MS2 system.^5–7^ In fluorophore-aptamer systems, specific RNA sequences are designed to bind fluorogenic organic molecules with high affinity,^3^ like the “vegetables” ^3,8–13^ and fluorescent light-up aptamer systems.^3,14–16^ Another strategy for RNA visualization exploits the tight binding of specific RNA sequences to organic fluorescence quenchers. By coupling a quencher molecule covalently to a fluorophore, RNA binding leads to dequenching and fluorescence intensity turn-on like in the SRB-2 system and RhoBast.^15,16^ Together, exploiting binding of a short RNA tag to a fluorescent molecule yields a fluorescence signal that reports on the tagged RNA in several RNA tagging platforms designed for live RNA tagging and tracking applications.

The Riboglow platform^17–19^ is an orthogonal RNA imaging system with unique photophysical properties. Riboglow is based on a short bacterial riboswitch RNA sequence that binds the ligand cobalamin (Cbl) with nM affinity.^17,20^ Cbl has fluorescence quenching properties.^17,21,22^ In Riboglow, a synthetic fluorophore is attached to Cbl via a chemical linker, and the proximity of Cbl reduces probe fluorescence.^17^ Fluorescence intensity and fluorescence lifetime increase upon RNA ligand binding to the probe (Figure 1A).^17,23,24^ It is noteworthy that varying the RNA Tag sequence that binds Cbl and presumably folds into a different conformation when bound to Cbl modulates the fluorescence lifetime increase (see also below), such that binding the same probe via different RNA Tag sequences may offer RNA multiplexing capabilities.^18^ The RNA Tag sequence we use here almost exclusively (the “RNA Tag”, Table S1) is called the “A tag” or “*env8*” elsewhere.^17,20,23–25^

**Figure 1:**
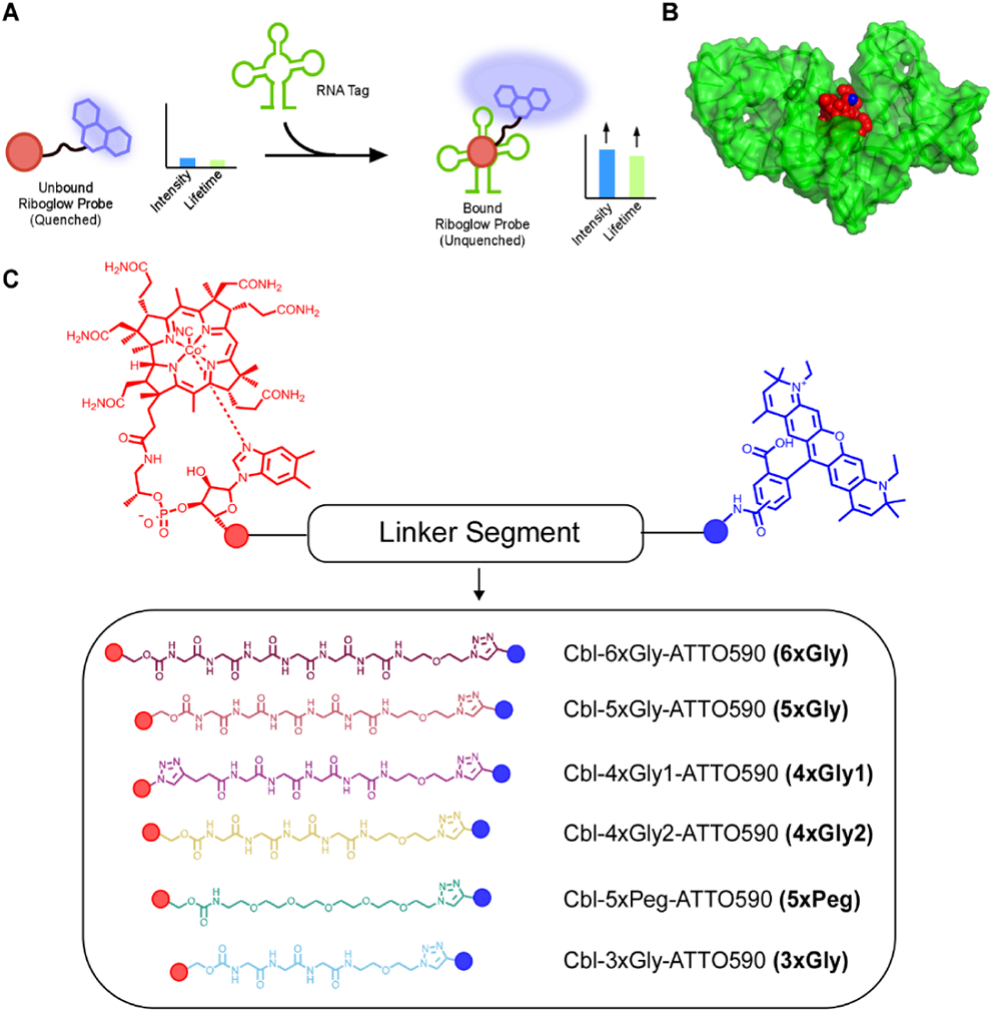
(A) Principle of the Riboglow platform. The cobalamin (Cbl) portion (red circle) of the Riboglow probe binds the RNA tag (green), separating the Cbl quencher from the covalently attached fluorophore, and fluorescence intensity and fluorescence lifetime increase. (B) Crystal structure of the Cbl riboswitch regulatory element (here called RNA Tag, green) with bound Cbl ligand (red) (PDB ID: 4FRN).^20^ Blue: 5′OH where the linker and fluorophore are attached to Cbl in all Riboglow probes. (C) Riboglow linker series for this study. Cbl (red) is covalently coupled to a fluorophore (blue), via a variable chemical linker.

Having the fluorophore attached to Cbl in Riboglow probes via the ribose 5′OH (Figure 1B, blue) affects the binding affinity to the RNA Tag minimally.^17^ While both probe fluorescence intensity and fluorescence lifetime increase upon RNA Tag binding,^17,18,23,24^ we found that cellular contrast is more robust with fluorescence lifetime imaging microscopy (FLIM) than fluorescence intensity-based imaging.^18^ This is likely because changes in probe and RNA concentrations that are hard to control in live cells are less critical for FLIM measurements. Previous studies with Riboglow probes Cbl-4xGly-ATTO590 (called 4xGly1 here, Figure 1C) and Cbl-5xPeg-ATTO590 (5xPeg, Figure 1C) revealed that the chemical linker affects probe fluorescence properties.^23^ For example, evaluating linker lengths in a series of ATTO488-containing probes revealed that a shorter linker increases probe quenching, indicating that FRET may at least partially contribute to probe quenching/dequenching.^17,23^ Together, a systematic assessment of the relationship between Riboglow linker chemistry and probe fluorescence intensity and lifetime is needed for detailed insights in photophysical properties of Riboglow to achieve the long-term goal of rational probe designs.

Here, we varied the chemical linker for our previous Riboglow probe (4xGly1, Figure 1C) systematically, evaluated fluorescence properties of all probe/RNA pairs, and characterized probe multiplexing capabilities. Cellular RNA visualization was evaluated in live mammalian cells for new probes. Together, insights from this work will guide future rational probe designs.

## Results and Discussion

We synthesized a series of four Riboglow probes with linkers of variable lengths (3xGly–6xGly, Figure 1C). As a general strategy, N-Boc-Gly-Gly-Gly was reacted with azido-PEG1-amine for late-stage installation of ATTO590 (Figure S1). Successive N-terminal elongation with either Boc-Gly-OSu or Boc-Gly-Gly-Gly-OSu yielded linkers of the required lengths. Following linker synthesis, a (1,2,4)-triazole-derivative of cyanocobalamin was added to the N-terminus of the linker, linking the two via a proximal carbamate.^26^ Finally, an alkyne-modified ATTO590 molecule was added to the C-terminal end of the Cbl-linker intermediate via a click reaction, forming a (1,2,3)-triazole. The chemical identities of all new Riboglow probes were verified via HRMS and purified to >95% as measured by HPLC (SI, HPLC Chromatograms). Importantly, the new Riboglow probes developed here (3xGly, 4xGly2, 5xGly, and 6xGly) have a proximal carbamate connecting the linker and Cbl (Figure 1C). The 5xPeg probe previously developed^17^ similarly contains the carbamate group, while the previously developed 4xGly1 probe has a Cbl-proximal (1,2,3)-triazole in addition to the triazole attaching the linker to ATTO590 (Figure 1C).^17^ Following synthesis, linker contour length and RMS linker end-to-end distances were estimated computationally by polymer chemistry approaches (listed in Table S2). Together, we built a series of Riboglow probes with variable Gly linkers sharing a common fluorophore to systematically investigate the photophysics of RNA/probe interactions.

To characterize probe fluorescence properties, we first collected unbound probe absorption spectra (Figure S2) and fluorescence intensities of all probes relative to free ATTO590 (Figure 2A,B). In line with previous observations,^17,23,24^ fluorescence of unbound probes was significantly lower than the fluorescence intensity of the free fluorophore, ATTO590. Overall, the probe fluorescence intensity was less than 13% of the intensity of free ATTO590 (Figure 2B, Table S3). Probe 3xGly had the lowest unbound fluorescence intensity of all probes measured (5.3% of free ATTO590), while 4xGly1 was the least quenched (12.1% of free ATTO590). Each additional Gly unit within the Gly series reduces unbound probe quenching (Figure 2B, Table S3), but 4xGly1 yields higher fluorescence signal than expected from this trend alone. The observation that increasing the linker length and therefore distance between Cbl (the quencher) and the fluorophore reduces probe fluorescence quenching indicates FRET quenching between Cbl and fluorophore. Unbound 5xPeg and 5xGly have comparable fluorescence intensities (10.0% and 10.8%, respectively) despite 5xGly being significantly longer than 5xPeg. 5xPeg and 4xGly1 both deviate from quenching trends predicted by linker length, suggesting a contribution of the linker’s architecture for fluorescence behavior.

**Figure 2:**
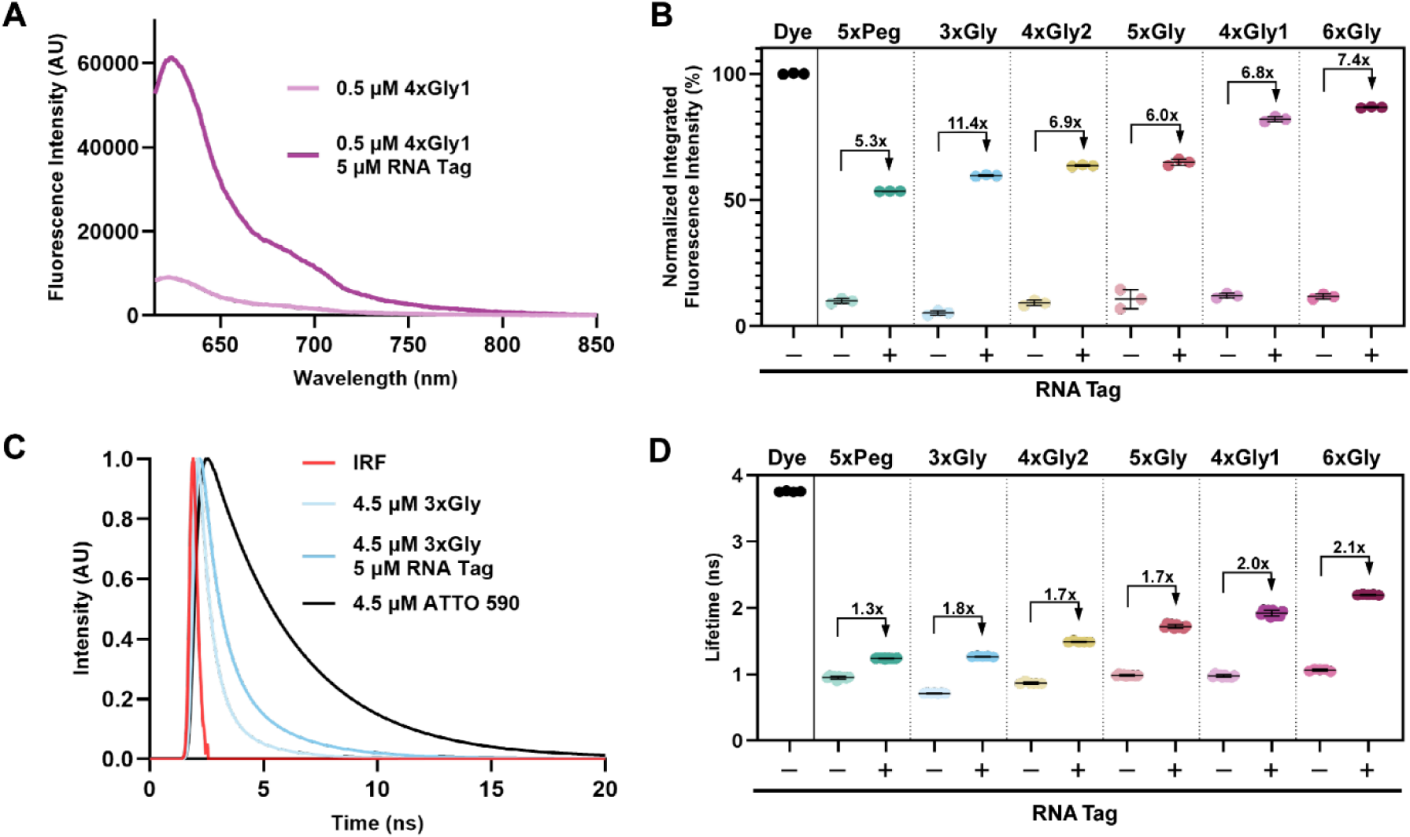
In vitro assessment of probe fluorescence. (A) Representative fluorescence intensity spectra of 0.5 μM 4xGly1 in the presence and absence of 5 μM purified RNA Tag. (B) Fluorescence intensity spectra of each sample in the presence and absence of the RNA tag were integrated, yielding one point (*n* = 3 independent experiments per sample). (C) Representative fluorescence lifetime decay curves for probe 3xGly in the presence and absence of the RNA Tag. Red: Instrument response function (IRF), black: decay curve of free ATTO590. (D) Fluorescence lifetime of Riboglow probes in the presence and absence of the RNA Tag. The absolute lifetime increase is listed in Supplementary Table 4. Each data point represents a technical replicate taken across multiple experiments with different RNA samples (*n* ≥ 15 technical replicates and ≥3 independent experiments per experimental group, see Figure S3).

Next, we evaluated fluorescence intensity of each ATTO590-containing probe in the presence of purified RNA ligand, the RNA Tag (Figure 2A,B). Overall, we observed probe dequenching in the presence of the RNA Tag, i.e., probe fluorescence intensity increased (Figure 2B). This observation is in line with previous work,^17,18,23^ suggesting that RNA Tag binding to Cbl sterically separates Cbl and the fluorophore in space, reducing fluorescence quenching. The fluorescence intensity of the RNA-bound probes increased with each additional Gly unit (Figure 2B, Table S3). Adding a Gly unit to 5xGly to yield 6xGly had the most substantial effect on probe dequenching. 4xGly1 had a greater RNA-bound intensity than 4xGly2 and 5xGly, suggesting a contribution of the additional triazole to probe dequenching upon RNA binding. Interestingly, 5xPeg had the lowest RNA-bound fluorescence intensity of all probes measured despite being less quenched than both 3xGly and 4xGly2. Fold turn-on of 3xGly upon RNA-binding was 11.4×, despite having a shorter linker than 5xPeg (Figure 1C, Table S2 and Table S3). The changes in relative probe fluorescence intensity upon RNA binding highlight the role of the probe linker chemistry in probe dequenching upon RNA-binding.

We next evaluated the fluorescence lifetime of all probes in the presence and absence of the RNA ligand. As in previous observations,^17,18,23^ all probes yield a reduced fluorescence lifetime compared with the lifetime of the free fluorophore (Figure 2D, Table S4). 3xGly had the lowest quenched lifetime (19.1% the lifetime of the free fluorophore) while 6xGly had the highest (28.4%). Each Gly unit increased the quenched lifetime by ∼0.1 ns (Figure 2D, Table S4). Interestingly, 5xPeg’s unbound lifetime was not significantly different from that of 5xGly, nor was the lifetime of 5xGly significantly different from 4xGly1 (Figure 2D). As with fluorescence intensity, the fluorescence lifetime of unbound Riboglow probes demonstrate linker length-dependent trends.

Upon adding the RNA ligand, the fluorescence lifetime of all probes increased substantially, as observed previously with other probes.^17,18,23^ Within the Gly linker series, we observed a correlation between the number of Gly residues and fluorescence lifetime, with probe lifetime increasing from 3xGly to 6xGly (Figure 2D, Table S4). Similar to fluorescence intensity trends, the greatest difference in increased fluorescence lifetime was between 5xGly and 6xGly (0.49 ns). This trend contrasts with the quenched lifetimes in the absence of the RNA ligand, where 3xGly and 4xGly2 had the greatest lifetime difference (0.16 ns). Probe 5xPeg breaks the length-related trend with the lowest dequenched lifetime of all probes measured. The lifetime turn-on of 5xPeg was the lowest of all probes (1.3x of its quenched lifetime), and its average fluorescence lifetime (∼1.24 ns) is not significantly different from that of 3xGly (1.27 ns) despite having a longer linker than both 3xGly and 4xGly2. For fluorescence lifetime turn-on, 4xGly1 once again disrupts length-related trends. Its dequenched lifetime (1.93 ns) is the second highest measured and it has a large lifetime turn-on (2.0× of its quenched lifetime), surpassed only by 6xGly. Importantly, all RNA-bound probes remain substantially quenched in comparison to the free fluorophore lifetime (3.76 ns). 6xGly has the greatest increase in fluorescence lifetime upon RNA binding (1.13 ns), which is only 42% of free ATTO590's fluorescence lifetime. The fluorescence lifetime data highlight the importance of Riboglow linker composition for fluorescence readouts when bound to the RNA.

Interestingly, increasing the linker length affects probe fluorescence readout in its RNA-bound state more than its unbound state (Figure 2B,D). We speculate that the interaction of Cbl and fluorophore through space is similar for all probes with Gly linkers in the absence of an RNA ligand, whereas probe binding to the RNA ligand yields conformations of the linker that are more linear in space. Such linearized linkers might affect differences of Cbl and fluorophore distance and/or orientation, causing a more pronounced effect on quenching.

An outlier to the length-related trends is 5xPeg which, despite being approximately the same length as 4xGly2, has a significantly lower fluorescence intensity and lifetime turn-on than all other probes tested (Figure 2B,D). Interestingly, fluorescence of the unbound 5xPeg fits into length-based trends and only deviates when bound to the RNA ligand. For example, 4xGly2 and 5xPeg have linkers of similar lengths and comparable unbound lifetimes (0.87 and 0.95 ns, respectively). In contrast, binding to the RNA ligand increases the difference in fluorescence lifetime between the two (1.46 ns and 1.24, respectively). A major structural difference between 5xPeg and the Gly probes is the restricted rotation around the amide bond. Previous work suggests that Peg linkers act as random coils and long Peg linkers may compress and contract in solution.^27^ In contrast, dequenching Riboglow demands maximum distance between Cbl and fluorophore, which may be best accomplished by the rigidity of peptide-based glycine linkers.

To further understand if differences in fluorescence turn-on correlate with binding to RNA, we measured RNA/probe binding affinities. Traditional binding measurements by isothermal titration calorimetry indicated that Cbl and a select Riboglow probe bind the RNA Tag with comparable affinity in the nM range (Table S5),^17,23,25,28^ but the necessity of large probe quantities is limiting for assessing RNA-binding affinities of our probe series in this way. Hence, we used probe fluorescence induction upon RNA-binding to determine the binding affinity (*K*_D_) of RNA and Riboglow probes (Figure 3A,B). All binding affinities were between 30 and 200 nM, similar to previous measurements (Figure 3C, Table S5).^17,23,25^ In contrast with fluorescence properties, the connection between linker construction and binding affinity is less defined, and the *K*_D_ values fluctuate without length-dependent trends. 4xGly1 had the lowest *K*_D_ at 35 nM, followed closely behind by 5xPeg and 6xGly (64 and 87 nM, respectively, Table S5). 4xGly2 had a *K*_D_ of 97 nM, while 5xGly and 3xGly had the highest *K*_D_s (149 and 199 nM, respectively). Interestingly, 4xGly1 and 5xGly, which are approximately the same length, have different *K*_D_s. Together, while we observed quantifiable binding affinity differences, all Riboglow probes yield submicromolar *K*_D_ values, independent of the probe’s architecture.

**Figure 3:**
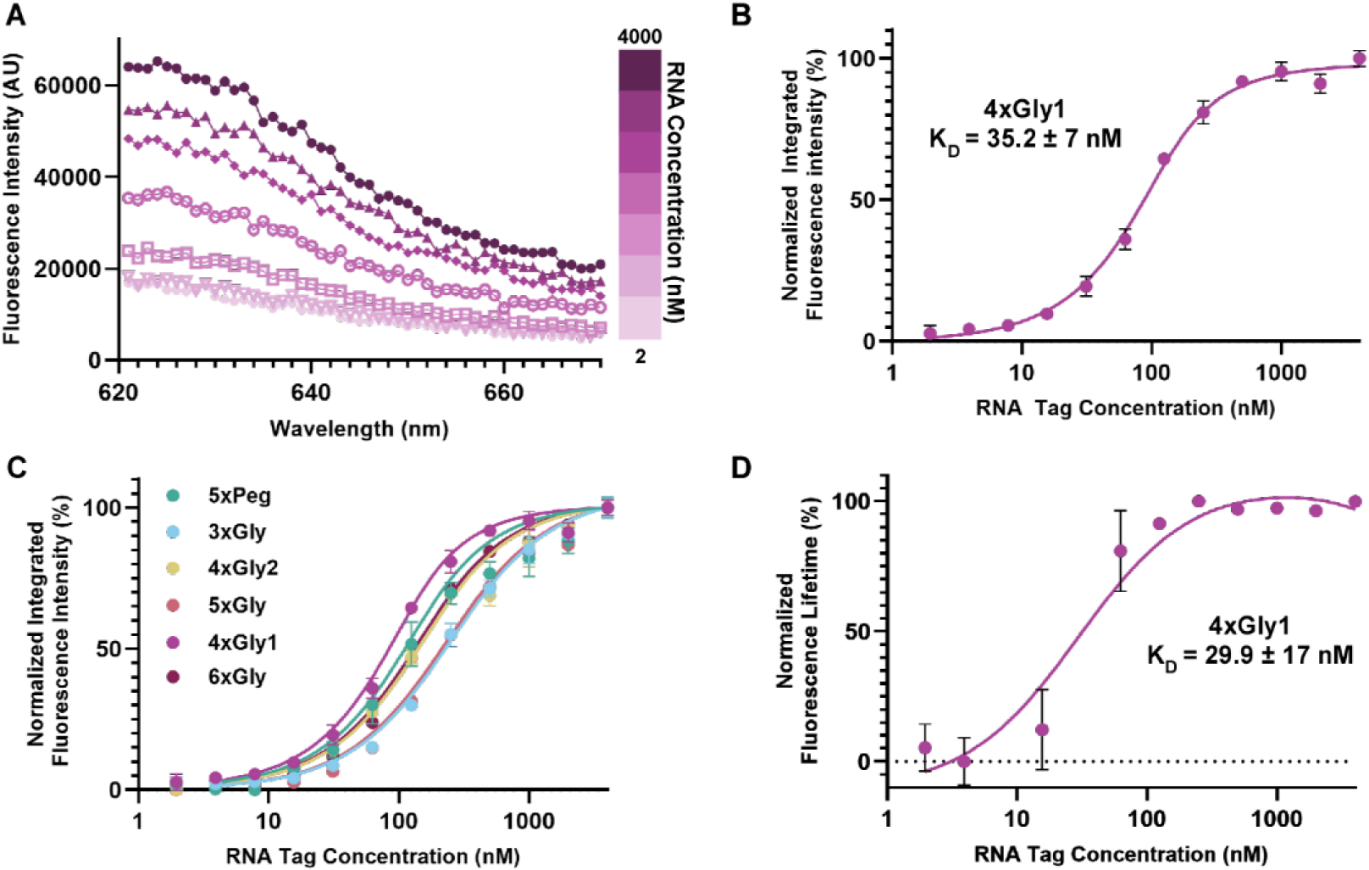
Riboglow probe binding to the RNA Tag via fluorescence induction. (A) Fluorescence intensity of 100 nM 4xGly1 with varying concentrations of the RNA Tag. (B) Determination of *K*_D_ by fluorescence induction. Each point is the mean of a technical triplicate of normalized integrated fluorescence of 4xGly1 with varying RNA Tag concentrations (from A). (C) RNA-binding for all Riboglow probes (*K*_D_ values: Table S5). (D) Determination of *K*_D_ using fluorescence lifetime. Each point is the mean of a technical quintuplicate of acquired fluorescence lifetime of 4xGly1 in the presence of different RNA Tag concentrations.

We then measured RNA Tag binding to the probe via fluorescence lifetime to determine if fluorescence lifetime can also report on binding affinity. We chose 4xGly1 as a model probe due to its large dynamic range of fluorescence lifetime turn-on in the presence of the RNA Tag (Figure 2D). The *K*_D_ measured by fluorescence lifetime increase was 29.9 nM, in line with the *K*_D_ determined by fluorescence intensity increase (Figure 3B,D). Together, we conclude that fluorescence lifetime increase and fluorescence intensity increase are both reliable techniques to measure the binding affinity of Riboglow probes.

Because the linker architecture of our probes affects fluorescence turn-on upon RNA binding, we hypothesized that chemical interactions of the RNA/probe complex may be modulated by varying the RNA ligand. We compared two RNA ligands that vary in sequence but bind Riboglow probes with nM affinity (A tag, D tag).^17,24,25^ We found previously that the fluorescence lifetime of 4xGly1 bound to the RNA A tag (the “RNA Tag” for all experiments above) and the RNA D tag were significantly different from one another both in vitro and in cells, enabling multiplexing of Riboglow where different RNA tags bind the same probe but induce differentiable fluorescence lifetime signals.^17,18,24^ We measured fluorescence lifetimes of all Riboglow probes in the presence of the RNA A tag and RNA D tag (Figure 4). As expected, the lifetime of 4xGly1 differed when bound to the A tag vs D tag by almost 0.2 ns, what we had found to be sufficient lifetime contrast before.^18^ Probes 3xGly and 5xGly also had unique lifetimes for each RNA tag, but these lifetimes were less distinguishable than those produced by 4xGly1 (0.1 and 0.04 ns difference for 3xGly and 5xGly, respectively). 3xGly produces a higher lifetime when bound to the RNA D tag, which deviates from trends for 5xPeg, 5xGly, and 4xGly1. Interestingly, the lifetimes of two probes, 4xGly2 and 6xGly, were not significantly different from one another, suggesting that these probes cannot be used to differentiate these RNA tags in multiplexing experiments (Table S6). These data reveal that linker architecture facilitates or forbids lifetime multiplexing capabilities.

**Figure 4:**
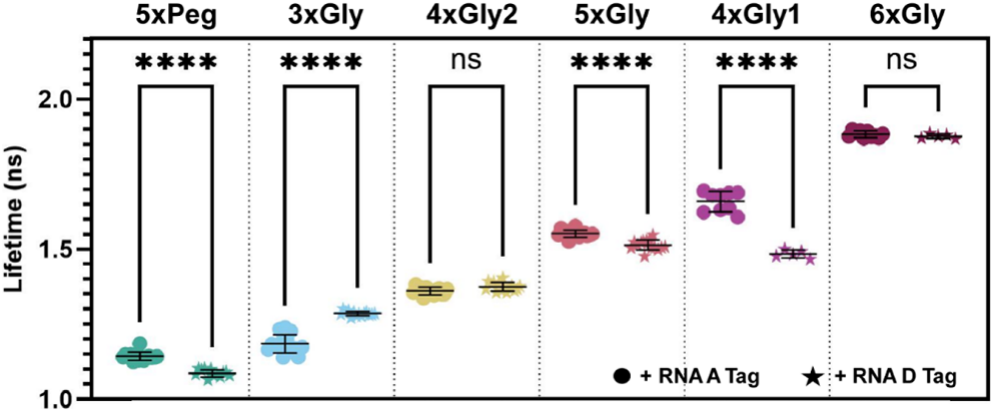
Fluorescence lifetime of all Riboglow probes bound to the RNA A and RNA D tags. One-way ANOVA (95% confidence limit); post hoc test (Tukey HSD). Each data point represents a technical replicate taken across multiple experiments with different RNA samples (*n* ≥ 5 technical replicates and ≥2 imaging sessions per experimental group).

A consistent observation in this study is the deviation of 4xGly1 from length-related trends present in the Gly-linker series. For instance, RNA binding to 4xGly1 results in significantly greater fluorescence turn-on than binding to 5xGly, despite having a linker of similar length (Figure 5B, Table S2). A key structural difference between 4xGly1 and 5xGly is the Cbl-proximal triazole in 4xGly1, which is absent in all other probes (Figure 5A). We speculate that a connection between this functional group, RNA binding, and fluorescence may exist. Notably, the Cbl-proximal triazole linkage reduces flexibility between Cbl and fluorophore due to the triazole ring structure compared with the more flexible carbamate linkage (Figure 1C). We observed a significantly lower *K*_D_ for 4xGly1 when bound to the RNA Tag vs 5xGly (Figure 3C, Figure 5C), indicating that tighter binding for 4xGly1 due to the proximal triazole may enable additional interaction with the RNA ligand. Additional rigidity provided by the triazole may also contribute to fluorescence observations. Together, the Cbl-proximal triazole in 4xGly1 positively influences the fluorescence turn-on upon RNA binding in comparison to linkers of similar length (Figure 2B,D, Figure 5B).

**Figure 5:**
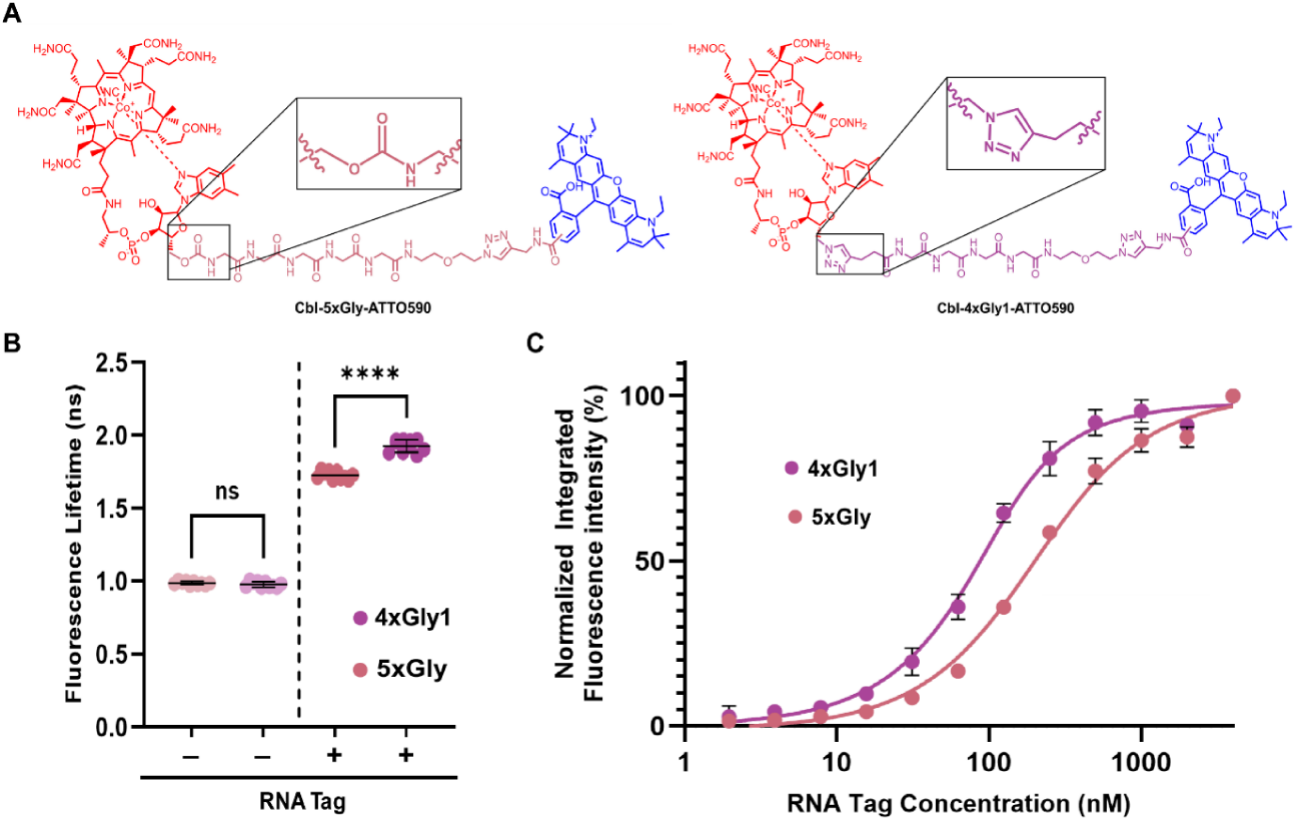
Comparison of Riboglow probes 5xGly and 4xGly1. (A) Chemical structures of 5xGly (left) and 4xGly1 (right). Zoom-in box shows the different functional group proximal to the Cbl-linker interface. (B) Fluorescence lifetime of 5xGly and 4xGly1 with and without the RNA Tag (5 μM). One-way ANOVA (95% confidence limit); post hoc test (Tukey HSD). Each data point represents a technical replicate taken across multiple experiments with different RNA samples (*n* ≥ 15 technical replicates and ≥3 independent experiments per experimental group). (C) Binding of RNA Tag to 5xGly and 4xGly1.

To test if in vitro lifetime trends are preserved in a cellular context, we measured cellular lifetimes of U-2 OS cells loaded with Riboglow probes in the presence and absence of β-Actin mRNA tagged with Riboglow RNA A tag (Figure 6). As expected, all three Riboglow probe lifetimes were significantly increased in the presence of tagged mRNA. Interestingly, the average cellular lifetime of 5xPeg is comparable with the lifetime measured in vitro, while both 4xGly1 and 6xGly show elevated cellular lifetimes compared with in vitro lifetimes in the absence of RNA A tag (Figure 6B, Table S4 and Table S7). Cellular environment also had a marked effect on the lifetime difference between the unbound and bound states for 4xGly1 and 6xGly. Despite their relatively large in vitro lifetime turn-on (0.95 and 1.13 ns for 4xGly1 and 6xGly, respectively), these probes had a greatly reduced lifetime turn-on in cells, at only ∼0.30 ns for both probes. Together, we find that the cellular environment may affect the conformation of Gly-containing linkers differently than the conformation of a Peg linker, resulting in less quenching of Gly-linkers, perhaps due to more steric separation due to more elongated conformations of Gly-linkers in the context of live cells vs a Peg linker.

**Figure 6:**
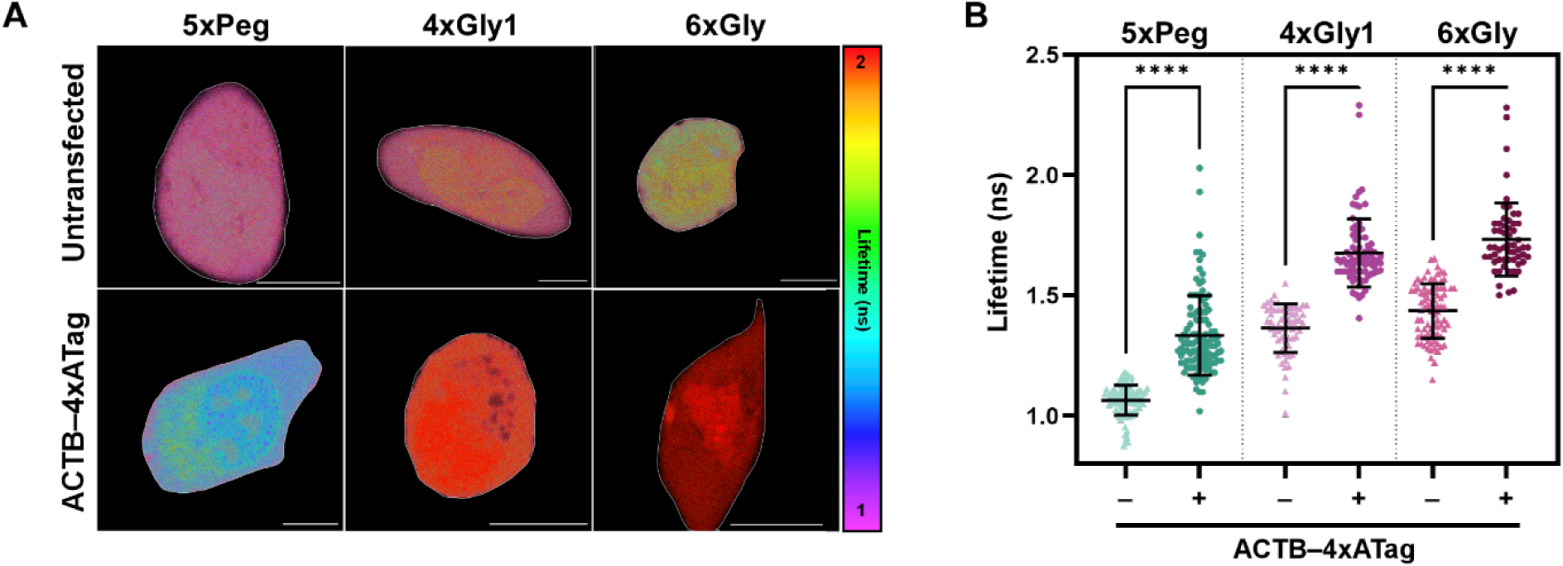
Lifetime of Riboglow probes in U-2 OS cells. Cells ± RNA A tagged to β-Actin mRNA were imaged by FLIM. (A) Representative cell images (Scale bar = 10 μm). Whole cell amplitude-weighted average lifetime for each cell was extracted and plotted in B. (B) Cellular lifetime turn-on for select Riboglow probes. One way ANOVA, *p* < 0.0001, *R*² = 0.7527; Tukey Multiple Comparisons *P* < 0.0001 (****) for all comparisons shown. 6xGly (−), *n* = 85 cells, x̅ = 1.436, SD = 0.1129; 6xGly (+), *n* = 57 cells, x̅ = 1.732, SD = 0.1516; 5xPeg (−), *n* = 100 cells, x̅ = 1.065, SD = 0.06201; 5xPEG (+), *n* = 113 cells, x̅ = 1.335, SD = 0.1646; 4xGly1 (−), *n* = 63 cells, x̅ = 1.365, SD = 0.1007; 4xGly1 (+), *n* = 80 cells, x̅ = 1.677, SD = 0.1420.

Together, we uncovered a surprising connection between linker architecture and fluorescence properties of fluorophore-linker probes in the Riboglow platform. By comparing 5xPeg to the glycine linker series, we identified the benefit that more rigid peptide linkers have over a Peg linker for probe dequenching. We also identified a privileged site for diversification and the unique function a proximal triazole gives to 4xGly1 in fluorescence dequenching and multiplexing, educating future Riboglow linker designs.

## Methods

### Riboglow Probes

Riboglow probes 5xPeg and 4xGly1 were a gift from Amy Palmer (UC Boulder). Synthesis and characterization of additional probes are described in Supporting Information. All probe stocks were brought up in DMSO and diluted to working stocks in PBS.

### UV–Vis Absorbance and Quantum Yield Determination

Probe UV–vis absorption measurements were taken on a Cary 60 UV–vis spectrophotometer using an Agilent ultramicro quartz cuvette (1 mm path length). The spectrophotometer was blanked using 10 μL of RNA buffer (100 mM KCl, 10 mM NaCl, 1 mM MgCl_2_, 40 mM HEPES pH 8.0). Absorbance was measured with 10 μL samples of probe in buffer from 200–800 nm in 1 nm increments (Figure S2). Probe concentrations were determined using the extinction coefficients for ATTO590 provided by the manufacturer (ATTOTEC, ε = 120,000 L mol^–1^ cm^–1^, 594 nm).^16^ The quantum yield (*Q)* of the new Riboglow probes was determined using the published *Q* of ATTO590 (0.80, ATTOTEC, Table S8). Thirty μL samples of serially diluted Riboglow probes were loaded onto a 384-wellplate (Corning, black polystyrene, clear glass bottom), and their absorbance and fluorescence intensity were measured on an Agilent Synergy H1 microplate reader. Absorbance was measured at 594 nm, and the fluorescence emission spectra was measured and integrated between 604 and 850 nm in 1 nm increments. Absorbance vs integrated fluorescence intensity plots were created in GraphPad Prism, and the *Q* of each Riboglow probe was determined from the slopes of fitted curves (Figure S4, Table S8).

### Plasmid DNA Preparation

DH5α competent *E. coli* cells (Thermo Fisher Scientific) were transformed with plasmids pCMV-GFP (gift from Connie Cepko, Addgene Plasmid #11153), pACTB-(A)­4x (gift from Amy Palmer, Addgene Plasmid #112058), pUC19 (gift from Joachim Messing, Addgene Plasmid #50005),^29^ or pUC19 containing the RNA Tag sequence (Table S1) per manufacturer protocol and grown on LB (1% tryptone, 0.5% yeast extract, 1% NaCl in H_2_O by weight) supplemented with 1.5% agar (Fisher) and/or antibiotic (100 μg mL^–1^ Ampicillin, 50 μg mL^–1^ Kanamycin) as necessary. Plasmids were purified via Plasmid Midi Kit (Qiagen) then diluted to 1 μg/μL in TE buffer (10 mM Tris-HCl, pH 8.0, 1 mM EDTA).

### RNA Ligand Preparation

The RNA ligands (elsewhere referred to as “RNA Tag”, “RNA A”, “RNA D”, or “env8” ^17,18,23,25^ ) were purified analogous to methods previously described.^17^ The plasmid DNA segment encoding the RNA was amplified by PCR as previously reported^30,31^ and cleaned up with the Monarch PCR & DNA Cleanup Kit. Linearized DNA was used to transcribe RNA using the T7 High Yield RNA Synthesis Kit (NEB). Transcription products were run on a 1.5 mm 10% acrylamide gel, stained with GelRed, and visualized on a Bio-Rad GelDoc Go Imaging System. The RNA tag band was cut out, minced, and soaked in a 500 mM sodium acetate solution overnight. The gel solution was centrifuged at 5000 *×* *g* for 1 min, the supernatant was collected, and the RNA was precipitated with 1 volume of isopropanol. The RNA solution was pelleted by centrifugation at 18,000 *×* *g* for 15 min at 4 °C, washed with 70% ethanol, and dissolved in nuclease-free water. DNA and RNA concentrations were determined on a NanoDrop 2000c spectrophotometer using extinction coefficients 50 ng·cm/μL and 40 ng·cm/μL at 260 nm for DNA and RNA, respectively.

### In Vitro Fluorescence Intensity

All RNA dilutions were performed in an RNase-free hood. RNA ligand and probe were diluted in RNA buffer to final concentrations of 5 μM and 0.5 μM, respectively. 0.5 μM ATTO590 was used for intensity normalization, and RNA buffer was used for blank subtraction. Thirty μL of the prepared samples were loaded into a 384-wellplate (Corning) and incubated in the dark for 30 min at RT to equilibrate. The fluorescence intensity of all wells was measured (monochromators, 594 nm excitation, 615–850 nm emission in 1 nm steps) on a microplate reader (Agilent). Collected fluorescence intensity curves were blank subtracted and integrated over the collected wavelengths. Replicates of integrated fluorescence intensity were plotted in GraphPad Prism.

### In Vitro Fluorescence Lifetime

All in vitro FLIM samples were prepared by diluting purified RNA ligand and probe in RNA buffer to final concentrations of 5 μM and 4.5 μM, respectively. Samples were incubated in the dark at RT for 30 min before a 20 μL droplet was placed on a slide. Fluorescence lifetime turn-on data (Figure 2D) and fluorescence lifetime binding data (Figure 3D) were collected on an Abberior STED FLIM microscope (100× oil objective or 20× air objective) with a fixed imaging area of 512 × 512 pixels. Data were acquired using a PicoQuant TimeHarp 260 card. Data per frame was acquired with a diode pulsed laser of 40 MHz and excitation at 561 nm (Semrock Em01-R488/568 + SP01-633RU filter). For multiplexed data (Figure 4), droplets were imaged on a Nikon ECLIPSE Ti2 using a 20× (Nikon N Plan Apochromat) objective set to 512 × 512 scanning areas. Photons were collected using a 40 MHz pulsed laser at 560 nm (PicoQuant Sepia PDL 828, PicoQuant LCU). Time-correlated single photon counting (TCSPC) was performed with a multichannel event timer (PicoQuant MultiHarp 150) and a hybrid photomultiplier detector (PicoQuant PMA Hybrid Series). Data per frame were acquired until a total threshold of 10^4^ counts was reached for all in vitro experiments.

### FLIM Analysis

For all FLIM experiments, the acquired decay function of each pixel in the region of interest (whole frame from in vitro FLIM, a single cell for cell experiments) was analyzed via multiexponential reconvolution fitting while maximizing the fit to extract the average amplitude weighted lifetime (τ_avg amp_) in PicoQuant SymPhoTime 64 (by using either 2 or 3 parameters). Parameters of the fit were minimized while accounting for total photon counts and decay curve fit.^30^

### Fluorescence Intensity Binding Assays

Fluorescence intensity binding assays were prepared with variable final RNA ligand concentration (4000, 2000, 1000, 500, 250, 125, 62.5, 31.3, 15.6, 7.8, 3.9, 2, 0.00 nM) and constant 100 nM final probe concentration in a 30 μL solution in a 384-well plate (Corning) with a buffer only well for blank subtraction. The plate was wrapped in aluminum foil, and the samples were allowed to incubate at RT for 30 min. The fluorescence intensity of all wells was measured as described above (594 nm excitation, 620–670 nm emission, 1 nm steps), resulting in the integrated fluorescence intensity for each sample. Fluorescence intensity values were normalized to the largest mean integrated fluorescence intensity value then plotted against log ([RNA]) in GraphPad Prism. Triplicate curves were fitted to the quadratic binding eq 1:

1 $$Y=m+\left(n-m\right)\frac{\left(c+x+K\right)-\sqrt{{\left(c+x+K\right)}^{2}z-4cx}}{2c}$$

where *Y* is the normalized integrated fluorescence value, *m* is the lower fluorescence baseline, *n* is the upper fluorescence baseline, *x* is the RNA concentration, *c* is the concentration of the probe, and *K* is the *K*_D_.^25^ Calculated *K*_D_ values are reported as the averages of at least three replicates, each with three technical replicates.

### Fluorescence Lifetime Binding Assays

Twenty μL solutions were prepared in the same manner as the fluorescence intensity binding solutions described above. Each sample was loaded on a 35 mm imaging dish (Ibidi) and imaged on the Abberior STED FLIM microscope as described for the fluorescence lifetime turn-on data. The fluorescence lifetime of each sample was collected as described above and fit using the above quadratic binding equation to determine the *K*_D_ for binding.

### Cell Culture

Adherent U-2 OS cells were obtained from the Tissue Culture and Biobanking Shared Resource Facility (Georgetown University) and grown in Dulbecco’s Modified Eagle Medium (DMEM, Gibco) supplemented with 10% Fetal Bovine Serum (FBS, Cytiva Hyclone) by volume on 90 mm TC Dishes (Fisher) in a humidified incubator at 37 °C and 5% CO_2_. Cells were washed with phosphate buffered saline (PBS, Corning) and passaged with TrypLE (Gibco) according to manufacturer instructions, as necessary.

### Cell Transfection and Imaging Preparation

0.3 × 10^6^ cells were seeded in an imaging dish (μ-Dish 35 mm, high, glass bottom, Ibidi) and grown until cells reached between 50% and 70% confluency as visualized under an EVOS M5000 microscope (Invitrogen). Cells were transfected with 1 μg pACTB-(A)­4x and 1 μg pCMV-GFP as a transfection marker (assuming that cotransfection of both plasmids occurs in this manner in at least 94% of all cells, as quantified before^17^) using 7 μL TransIT-2020 (Mirus Bio) according to manufacturer instructions. 48 h post transfection, cells were bead loaded^31^ analogous to methods previously described^17,18^ with Riboglow fluorescent probe. Briefly, all media was removed, 3 μL of 15 μM probe was added, cells were loaded, media was added, and cells were let incubate for 10 min before being washed and covered in Fluorobrite (Gibco) supplemented with 10% FBS by volume. Cells were imaged within 3 h of loading.

### Cell Imaging

Cells were imaged by scanning confocal microscopy at 488 and 561 nm with the Nikon AX system described above for in vitro multiplexing data. Cells were imaged using a 20× or 100× objective set to 2048 × 2048 or 512 × 512 scanning areas. FLIM imaging was done as for in vitro samples (above). Briefly, cells expressing GFP as determined by excitation at 488 nm were imaged with the same microscope, objectives, and scanning area using a 40 MHz pulsed laser at 560 nm. Data per frame were acquired to maximize photon counts within the 3 h imaging time frame.

## Supplementary Material



## Notes

UV–vis absorption spectra, *K*_D_ values, fluorescence intensity and lifetime data, linker length calculations, multiplexing data, cellular lifetime data, quantum yield determination, HRMS and HPLC traces of Riboglow probes, and sequences of Riboglow RNA A and D tags; ^1^H NMR, ^13^C NMR, and HRMS provided for synthetic intermediates (PDF)
