# Supplementary material for: Evaluating Linker Architecture in RNA-Detecting Riboglow Probes and Effects on Fluorescence Turn-On
Source: ACS Chem Biol. 2026 Feb 2;21(2):371–9. doi: 10.1021/acschembio.5c00917 (PMC12930364; doi:10.1021/acschembio.5c00917)

Evaluating Linker Architecture in RNA-Detecting Riboglow Probes and Effects on Fluorescence Turn-On

Luke K. Shafik,[1] Gareth M. Francis,[1] Giulia Chitu,[1] Jenna Hanson,[2] Sebastian Lis,[2] Kiera Cunningham,[2] Brooke Tatarian,[2] Aaron R. Van Dyke*[2], Esther Braselmann*[1]

1. Georgetown University, 3700 O St. NW, Washington, D.C., 20057.
2. Fairfield University, 1073 N. Benson Rd, Fairfield, CT, 06824.

* Co-corresponding authors: avandyke@fairfield.edu, esther.braselmann@georgetown.edu

Table of Contents

Supplementary Figures and Tables.....2
Supplementary Figure 1.....4
Supplementary Figure 2.....5
Supplementary Figure 3.....6
Supplementary Figure 4.....7
Supplementary Table 1.....8
Supplementary Table 2.....9
Supplementary Table 3.....10
Supplementary Table 4.....11
Supplementary Table 5.....12
Supplementary Table 6.....13
Supplementary Table 7.....14
Supplementary Table 8.....15
Riboglow Probe Synthesis and Characterization.....16
General Procedure for Linker Synthesis.....16
Supporting Compound (1).....17
Supporting Compound (2).....18
Supporting Compound (3).....19
Supporting Compound (4).....20
Cobalamin Triazole.....21
General Procedure for Coupling Polyglycine Linker to Cyanocobalamin.....22
Supporting Compound (5).....23
Supporting Compound (6).....24
Supporting Compound (7).....25
Supporting Compound (8).....26
Riboglow Probes Synthesized in This Study.....27
General Procedure for Click Coupling ATTO590 Alkyne.....27
Cbl-3xGly-ATTO590 (3xGly).....28
Cbl-4xGly2-ATTO590 (4xGly2).....29
Cbl-5xGly-ATTO590 (5xGly).....30
Cbl-6xGly-ATTO590 (6xGly).....31
References.....32

HPLC Chromatograms……………………………………………………………………...33

Supporting Compound (5) 33

Supporting Compound (6) 34

Supporting Compound (7) 35

Supporting Compound (8) 36

Cbl-3xGly-ATTO590 (3xGly) 37

Cbl-4xGly2-ATTO590 (4xGly2) 38

Cbl-5xGly-ATTO590 (5xGly) 39

Cbl-6xGly-ATTO590 (6xGly) 40

^{1}H-NMR Spectra………………………………………………………….................41

Supporting Compound (1) 41

Supporting Compound (2) 42

Supporting Compound (3) 43

Supporting Compound (4) 44

^{13}C-NMR Spectra…………………………………………………………...............45

Supporting Compound (1) 45

Supporting Compound (2) 46

Supporting Compound (3) 47

Supporting Compound (4) 48

Supplementary Figures and Tables

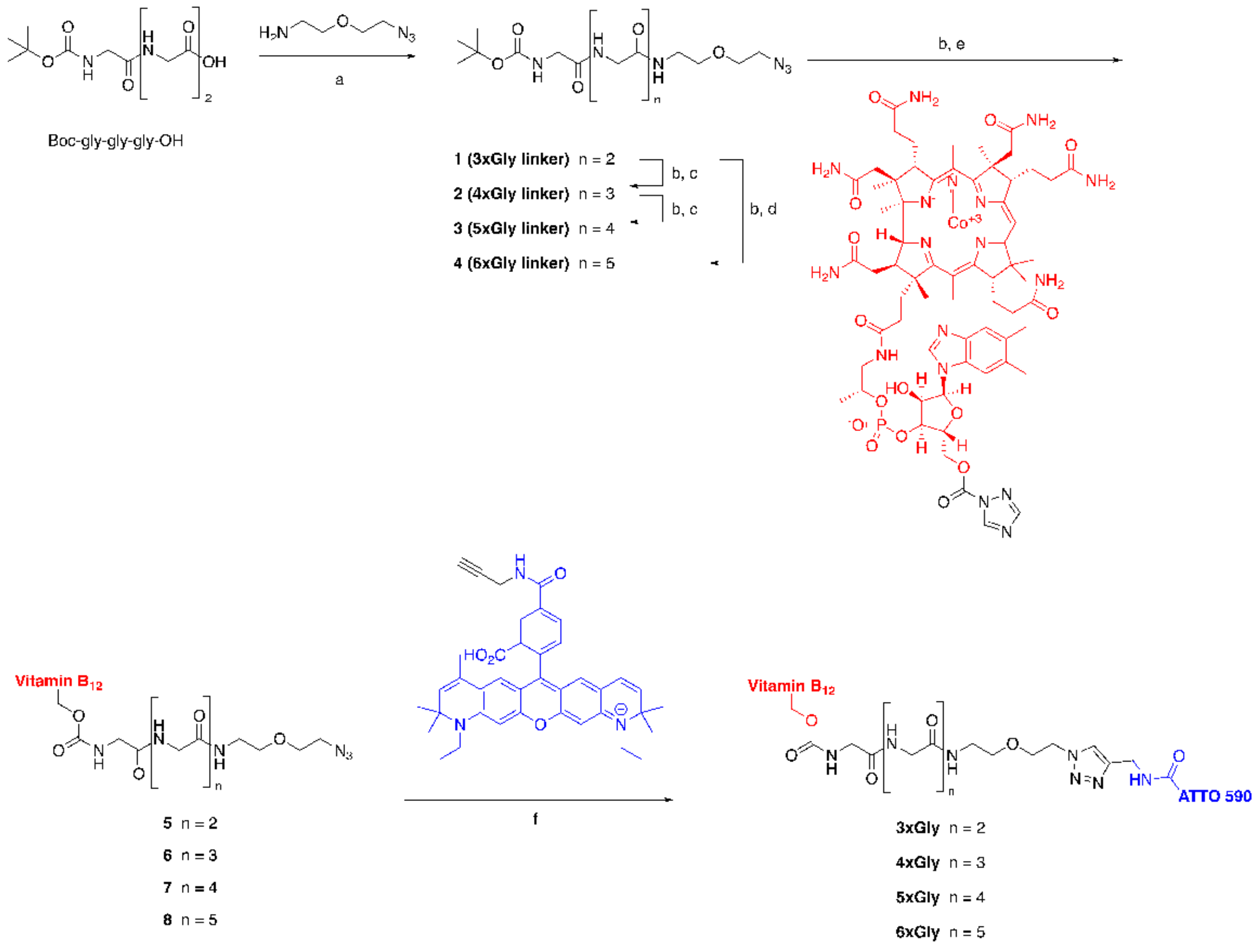

Supplementary Figure 1: Synthesis of Riboglow probes used in the study. Commercially available Boc-Gly-Gly-Gly-OH is iteratively elongated then coupled to Cbl followed by a click [3+2] azide-alkyne cycloaddition to ATTO 590: a) EDC•HCl, iPr_2NEt, DMF. b) TFA, DCM. c) Boc-Gly-OSu, iPr_2NEt, CH_3CN. d) Boc-Gly-Gly-Gly-OSu. e) **Vitamin B12** triazole, iPr_2NEt, DMSO. f) **ATTO 590** alkyne, CuI, TBTA, DMF, H_2O.

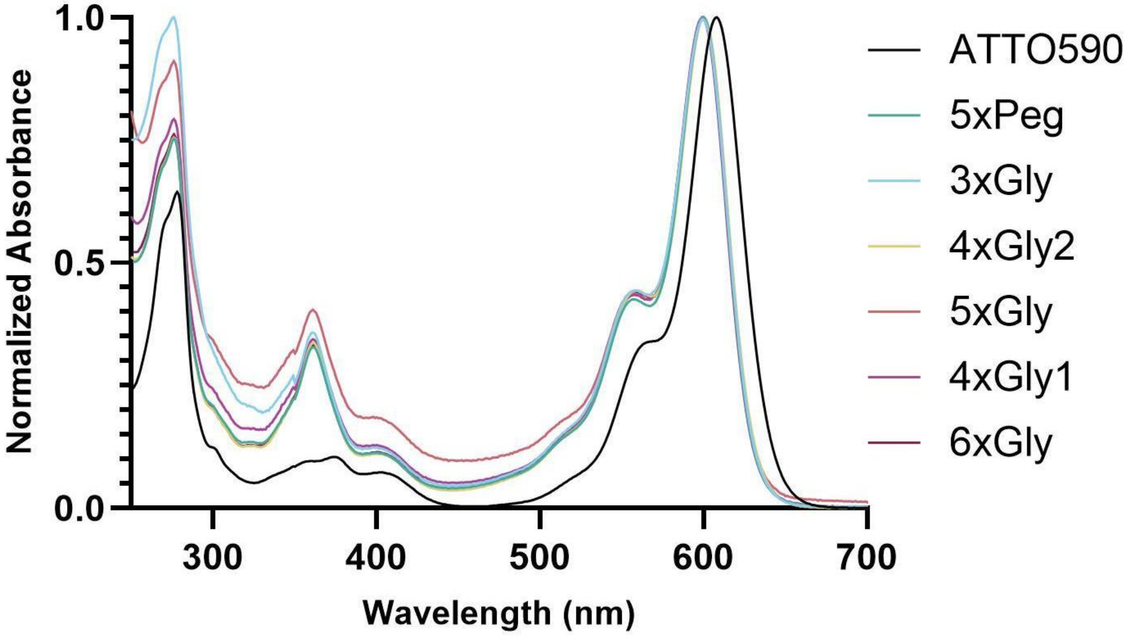

Supplementary Figure 2: Normalized UV-vis absorption spectra of all Riboglow probes included in this study. Absorption spectrum of ATTO 590 included in black.

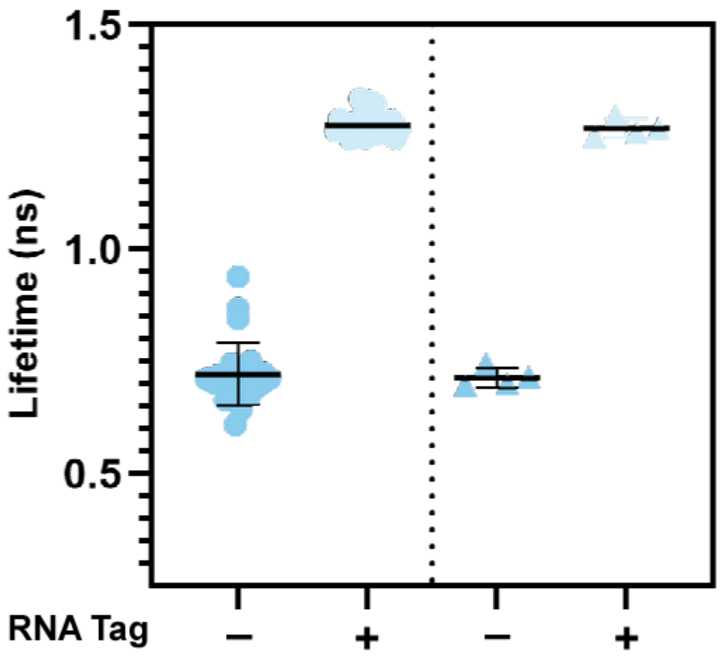

Supplementary Figure 3: Reproducibility of fluorescence lifetime measurements for Riboglow probe samples. In vitro fluorescence lifetimes of 3xGly are plotted as each experimental lifetime value extracted from a decay curve (main Fig. 2C) on the left (circles), and fluorescence lifetimes for a set of individual measurements was averaged for each experimental session on the right (triangles). Error bars: mean and standard deviation.

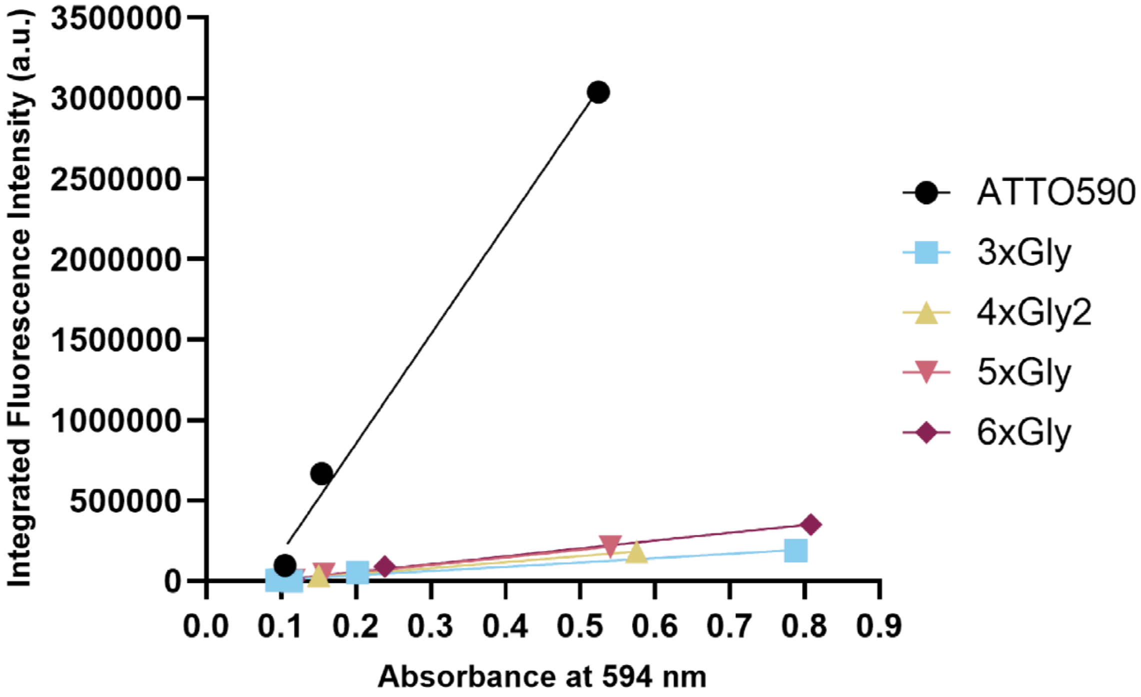

Supplementary Figure 4: Determination of the quantum yield for new Riboglow probes synthesized in this study. The absorbance and integrated fluorescence intensity was collected from a serial dilution of each sample. See Supplementary Table 8 for a summary of the determined quantum yield values.

Name	Sequence
RNA Tag (A tag)	5'- GGC CUA AAA GCG UAG UGG GAA AGU GAC GUG AAA UUC GUC CAG AUU ACU UGA UAC GGU UAU ACU CCG AAU GCC ACC UAG GCC AUA CAA CGA GCA AGG AGA CUC -3'
RNA D tag	5'- UAC UGA AAG CGU GGU GGG AAA CAA UGU GAA AGU CAU UGA CUG UUC CUG CAA CGG UAA GCG CUU CGG CGC GAG UCC GAA UGC CAC CCA GUA AAG UCC GCU GUC GAG UGA AGG CCA GGA AAA GUC UAA CUC A -3'

Supplementary Table 1: RNA sequences of the Riboglow RNA Tag (A tag) and RNA D tag previously developed.[1,2]

Linker	Contour Length (nm)	RMS End-to-End Distance (nm)
5xPeg	2.95	2.44
3xGly	2.72	2.64
4xGly2	3.12	2.85
5xGly	3.50	3.03
4xGly1	3.48	3.21
6xGly	3.89	3.20

Supplementary Table 2: Length calculations for linkers of all Riboglow probes used in this study. Linker lengths were estimated using monomer length to determine the maximum extended length (contour length) and using Flory radius calculations for the effective linker length, root-mean-square (RMS) end-to-end distance.[3–5]

Sample	% Fluorescence Intensity	Fold Turn-On
ATTO 590	100.0	N/A
5xPeg	10.0	5.3
5xPeg + RNA Tag	53.6	
3xGly	5.3	11.4
3xGly + RNA Tag	59.8	
4xGly2	9.2	6.9
4xGly2 + RNA Tag	63.8	
5xGly	10.8	6.0
5xGly + RNA Tag	65.1	
4xGly1	12.1	6.8
4xGly1 + RNA Tag	82.2	
6xGly	11.8	7.4
6xGly + RNA Tag	86.8	

Supplementary Table 3: Summary of fluorescence intensity data of Riboglow probes included in this study.

Sample	Fluorescence Lifetime (ns)	% Fluorescence Lifetime	Fold Turn-On	Lifetime Increase (ns)
ATTO 590	3.76	100.0	N/A	N/A
5xPeg	0.95	25.4	1.3	0.29
5xPeg + RNA Tag	1.24	33.1		
3xGly	0.72	19.1	1.8	0.56
3xGly + RNA Tag	1.27	33.8		
4xGly2	0.87	23.2	1.7	0.59
4xGly2 + RNA Tag	1.49	39.8		
5xGly	0.99	26.1	1.7	0.72
5xGly + RNA Tag	1.73	45.3		
4xGly1	0.98	26.0	2.0	0.95
4xGly1 + RNA Tag	1.93	51.3		
6xGly	1.07	28.4	2.1	1.13
6xGly + RNA Tag	2.20	58.6		

Supplementary Table 4: Summary of fluorescence lifetime data of Riboglow probes included in this study.

Study	Ligand	RNA	Method	K_D (nM)
Braselmann et al. (2018)[1]	Cbl	RNA A tag	ITC	37 ± 1
		RNA D tag	ITC	2.2 ± 1.6
	Cbl-5xPeg-ATTO590	RNA A tag	ITC	34 ± 9
		RNA D tag	ITC	3.0 ± 0.6
Lennon et al. (2022)[6]	Cbl-5xPeg-ATTO590	RNA A tag	FIBA	28 ± 7
Sarfraz et al. (2024)[7]	CNCbl	RNA A tag	ITC	99 ± 30
This study	Cbl-5xPeg-ATTO590	RNA A tag	FIBA	64 ± 20
	Cbl-3xGly-ATTO590	RNA A tag	FIBA	199 ± 26
	Cbl-4xGly2-ATTO590	RNA A tag	FIBA	97 ± 29
	Cbl-5xGly-ATTO590	RNA A tag	FIBA	149 ± 22
	Cbl-4xGly1-ATTO590	RNA A tag	FIBA	35 ± 7
			FLBA	30 ± 17
	Cbl-6xGly-ATTO590	RNA A tag	FIBA	87 ± 13

Supplementary Table 5: K_D values of Riboglow probes as determined in this study and previous literature. Methods for K_D calculation are isothermal titration calorimetry (ITC), fluorescence intensity binding assays (FIBA), or fluorescence lifetime binding assays (FLBA). Mean values plus/minus standard deviation for at least 3 independent experiments are listed. Ligand names for probes are listed as the detailed name (see main Fig. 1C).

Sample	Average Lifetime (ns)	Lifetime Difference (ns)	P-value
5xPeg + RNA A tag	1.14	0.06	<0.0001
5xPeg + RNA D tag	1.09		****
3xGly + RNA A tag	1.19	0.10	<0.0001
3xGly + RNA D tag	1.29		****
4xGly2 + RNA A tag	1.36	0.01	0.6477
4xGly2 + RNA D tag	1.37		ns
5xGly + RNA A tag	1.55	0.04	<0.0001
5xGly + RNA D tag	1.51		****
4xGly1 + RNA A tag	1.66	0.18	<0.0001
4xGly1 + RNA D tag	1.48		****
6xGly + RNA A tag	1.88	0.01	0.9998
6xGly + RNA D tag	1.88		ns

Supplementary Table 6: Overview of in vitro lifetimes of Riboglow probes bound to the RNA A and D tags. P-values of RNA A and D tag comparisons are also shown (One-way ANOVA, 95% confidence limit).

Probe	U2-OS Condition	Average Lifetime (ns)	Lifetime Difference (ns)	P-value
5xPeg	Untransfected	1.06	0.27	<0.0001
	ACTB-4xATag	1.33		****
4xGly1	Untransfected	1.36	0.31	<0.0001
	ACTB-4xATag	1.68		****
6xGly	Untransfected	1.44	0.30	<0.0001
	ACTB-4xATag	1.73		****

Supplementary Table 7: Overview of cellular lifetimes of Riboglow probes in U2-OS cells. Cells were either untransfected or transfected with a plasmid to produce beta-actin mRNA tagged with four repeats of the RNA Tag (ACTB-4xATag). P-values of comparisons between untransfected and transfected cells for each probe are also shown (One-way ANOVA, 95% confidence limit).

Sample	Quantum Yield	Source
ATTO 590	0.80	ATTO-TEC
3xGly	0.03	this study
4xGly2	0.04	this study
5xGly	0.06	this study
6xGly	0.06	this study
5xPeg	0.06	Braselmann et al. (2018)[1]
4xGly1	0.09	Braselmann et al. (2018)[1]

Supplementary Table 8: Quantum yield calculations for all new Riboglow probes synthesized in this study.

Riboglow Probe Synthesis and Characterization

General Information

All reactions were performed under an oxygen-free atmosphere of argon with rigid exclusion of moisture from reagents and glassware. N,N-dimethylformamide (DMF), dimethyl sulfoxide (DMSO), acetonitrile (CH_3CN), and dichloromethane (DCM) were dried through activated alumina columns prior to use. High-performance liquid chromatography was performed using a Shimadzu LC20-AT and Agilent Zorbax 300SB-C8 reverse phase column (5 µm, 9.4 *x* 250 mm) with acetonitrile and 0.1% TFA in H_2O as the mobile phases. Microwave reactions were performed using a CEM Discover SP synthesizer. ^{1}H and ^{13}C spectra were recorded in DMSO, unless otherwise noted, on a 400 MHz MHz JEOL spectrometer. Chemical shifts in NMR spectra are reported in parts per million (ppm) on the δ scale from an internal standard of residual DMSO ^{1}H-NMR (2.50 ppm) ^{13}C-NMR (39.52 ppm). Data are reported as follows: chemical shift, integration, multiplicity (s = singlet, d = doublet, t = triplet, q = quartet, m = multiplet, app = apparent, and br = broad) and coupling constant(s).

Riboglow Probe Synthesis and Characterization

General Procedure for Linker Synthesis

Supporting Compound (1)

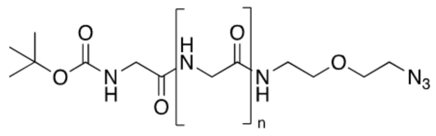

1 n = 2

Boc-GGG-OH (150 mg, 0.52 mmol) was dissolved in CH_3CN (4 mL) in a 10 mL microwave vial with a stir bar. Oxyma pure (81 mg, 0.57 mmol), iPr_2NEt (226 µL, 1.3 mmol) and dicyclohexylcarbodiimide (DCC) (112 mg, 0.54 mmol) were added sequentially. 2-(2-Azidoethoxy)ethanamine (0.62 mmol, 81 mg) was added last. The reaction vessel was sealed and heated to 80°C by microwave for 15 min with stirring. The resulting slurry was chilled on ice for 5 minutes. The white precipitate was removed by filtration with celite and rinsed with ice cold CH_3CN (5 mL). The solvent collected by filtration was removed by rotary evaporation. The resulting crude material was precipitated into ice cold Et_2O using a minimal amount of CH_3CN. The peptide was collected by centrifugation (3000 *x* g, 3 min, 4°C). The pellet was washed three times with Et_2O (30 mL). Residual solvent was removed in vacuo. The product (**1**) was isolated as a white powder (164 mg, 79% yield).

^{1}H-NMR (400 MHz, DMSO) δ: 1.37 (s, 9H), 3.20-3.24 (q, *J* = 5.9 Hz, 2H), 3.38-3.40 (t, *J* = 5.0 Hz, 2H), 3.43-3.46 (t, *J* = 5.9 Hz, 2H), 3.57-3.59 (m, 4H), 3.66-3.67 (d, *J* = 5.7 Hz, 2H), 3.72-3.73 (d, *J* = 5.5 Hz, 2H), 7.01-7.04 (t, *J* = 5.7 Hz, 1H), 7.83-7.86 (t, *J* = 5.3 Hz, 1H), 8.07-8.10 (d, *J* = 5.2 Hz, 1H), 8.10-8.13 (d, *J* = 5.2 Hz, 1H).

^{13}C-NMR (100 MHz, DMSO) δ: 28.2, 38.5, 41.9, 42.1, 43.3, 49.9, 68.7, 69.0, 78.1, 155.9, 168.8, 169.1, 169.9.

HRMS: HR-MS (ESI) Calcd for $C_{15}H_{27}N_7O_6$ $[M+H]^+$ 402.2095, found 402.2102.

Supporting Compound (2)

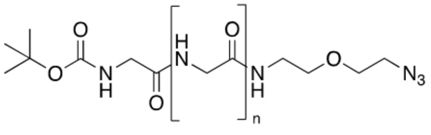

2 n = 3

Supporting compound (**1**) (433 mg, 1.08 mmol) was dissolved in DCM (10 mL). The mixture was cooled on ice to 0°C. TFA (3 mL) was added dropwise using a micropipette. The resulting mixture was stirred on ice at 0°C for an hour, and transferred to a falcon tube. A stream of air was used to evaporate the DCM. The residual oil was vortexed with H_2O (8 mL) and CH_3CN (2 mL). The product was lyophilized overnight and used without further purification in the next step.

The crude TFA•amine salt (324 mg, 0.78 mmol) was dissolved in CH_3CN (3.0 mL) and transferred to a microwave vial with iPr_2NEt (302 mg, 2.34 mmol) and stirred. Boc-Gly-OSu (255 mg, 0.94 mmol) was added to the microwave vial. The reaction vessel was sealed and heated to 80°C in a microwave reactor for 15 min. The product was transferred to a falcon tube and centrifuged (3000 *x* g, 3 min, 4°C). The supernatant was discarded. The pellet was washed with ice-cold ether (15 mL), vortexed until suspended, and centrifuged (3000 *x* g, 3 min, 4°C). The supernatant was discarded. This process was repeated two additional times. The final pellet was dissolved in water (10 mL) and CH_3CN (3 mL) and lyophilized. The product was isolated as a white solid and used without further purification (215 mg, 60% yield).

^{1}H-NMR (400 MHz, DMSO) δ: 1.38 (s, 9H), 3.21-3.25 (q, *J* = 5.5 Hz, 2H), 3.38-3.40 (t, *J* = 4.6 Hz, 2H), 3.43-3.50 (t, *J* = 5.7 Hz, 2H), 3.57-3.60 (m, 4H), 3.66-3.68 (d, *J* = 5.5 Hz, 2H), 3.72-3.75 (t, *J* = 6.0 Hz, 4H), 7.00-7.03 (t, *J* = 5.3 Hz, 1H), 7.84-7.87 (t, *J* = 4.6 Hz, 1H), 8.05-8.06 (t, *J* = 4.6 Hz, 1H), 8.10-8.11 (t, *J* = 5.0 Hz, 1H), 8.15-8.17 (t, *J* = 4.8 Hz, 1H).

^{13}C-NMR (100 MHz, DMSO) δ: 28.1, 38.5, 41.9, 42.0, 42.1, 43.3, 49.9, 68.7, 68.9, 78.1, 155.8, 168.7, 168.9, 169.3, 169.8.

HRMS: HR-MS (ESI) Calcd for $C_{17}H_{30}N_8O_7$ $[M+Na]^+$ 481.2129, found 481.2137.

Supporting Compound (3)

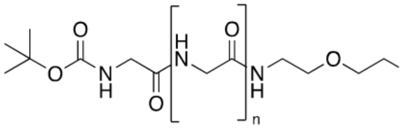

3 n = 4

Supporting compound (**2**) (322 mg, 0.704 mmol) was dissolved in DCM (10 mL). The mixture was cooled on ice to 0°C. TFA (3 mL) was added dropwise using a micropipette. The resulting mixture was stirred on ice at 0°C for an hour and transferred to a large falcon tube. A stream of air was used to evaporate the DCM. The remaining product was vortexed with H_2O (8 mL) and CH_3CN (2 mL). The product was lyophilized overnight and used without further purification in the next step.

The crude TFA•amine salt (332 mg, 0.704 mmol) was dissolved in CH_3CN (3.0 mL) and transferred to a microwave vial with iPr_2NEt (735 µL, 4.22 mmol) and stirred. Boc-Gly-OSu (228 mg, 0.84 mmol) was added to the microwave vial. The reaction vessel was sealed and heated to 80°C in a microwave reactor for 15 min. The product was transferred to a falcon tube and centrifuged (3000 *x* g, 3 min, 4°C). The supernatant was discarded. The pellet was washed with ice-cold ether (15 mL), vortexed until mixed, and centrifuged (3000 *x* g, 3 min, 4°C). The supernatant was discarded. This process was repeated two additional times. The final pellet was dissolved in water (10 mL) and CH_3CN (3 mL) and lyophilized. The product was isolated as a white solid and used without further purification (116 mg, 32% yield).

^{1}H-NMR (400 MHz, DMSO) δ: 1.38 (s, 9H), 3.21-3.25 (q, *J* = 5.7 Hz, 2H), 3.37-3.40 (t, *J* = 5.0 Hz, 2H), 3.43-3.46 (t, *J* = 6.2 Hz, 2H), 3.57-3.60 (m, 4H), 3.67-3.68 (d, *J* = 5.7 Hz, 2H), 3.72-3.77 (m, 6H), 7.00-7.03 (t, *J* = 5.7 Hz, 1H), 7.84-7.87 (t, *J* = 5.5 Hz, 1H), 8.04-8.21 (m, 4H).

^{13}C-NMR (100 MHz, DMSO) δ: 28.2, 38.6, 41.8, 42.1, 42.3, 43.0, 43.4, 49.9, 68.7, 69.1, 78.2, 155.9, 168.7, 168.8, 169.0, 169.3, 169.8.

HRMS: HR-MS (ESI) Calcd for $C_{19}H_{33}N_9O_8$ $[M+H]^+$ 516.2525, found 516.2519.

Supporting Compound (4)

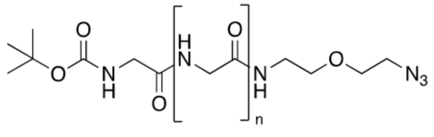

4 n = 5

Supporting compound (**1**) (433 mg, 1.08 mmol) was dissolved in DCM (10 mL). The mixture was cooled on ice to 0°C. TFA (3 mL) and water (0.5 mL) were added dropwise using a micropipette. The resulting mixture was stirred on ice at 0°C for an hour, and transferred to a large falcon tube. A stream of air was used to evaporate the DCM. The remaining product was vortexed with H_2O (8 mL) and CH_3CN (2 mL). The product was lyophilized overnight and used without further purification in the next step.

The crude TFA•amine salt (332 mg, 0.71 mmol) was dissolved in CH_3CN (3.0 mL) and transferred to a microwave vial with iPr_2NEt (742 µL, 4.26 mmol) and stirred. Boc-Gly-Gly-Gly-OSu was prepared as previously described in literature.[8] Boc-Gly-Gly-Gly-OSu (482 mg, 1.25 mmol) was added to the microwave vial. The reaction vessel was sealed and heated to 80°C in a microwave reactor for 15 min. The product was transferred to a falcon tube and centrifuged (3000 *x* g, 3 min, 4°C). The supernatant was discarded. The pellet was washed with ice-cold ether (15 mL), vortexed until mixed, and centrifuged (3000 *x* g, 3 min, 4°C). The supernatant was discarded. This process was repeated two additional times. The final pellet was dissolved in water (10 mL) and CH_3CN (3 mL) and lyophilized. The product was isolated as a white solid and used without further purification (253 mg, 57 % yield).

^{1}H-NMR (400 MHz, DMSO) δ: 1.38 (s, 9H), 3.20-3.24 (q, J = 5.6 Hz, 2H), 3.37-3.41 (t, J = 5.2 Hz, 2H), 3.43-3.46 (t, J = 6.2 Hz, 2H), 3.56-3.59 (m, 4H), 3.67-3.68 (d, J = 5.5 Hz, 2H), 3.70-3.76 (m, 4H), 3.76-3.79 (m, 2H), 7.00-7.03 (t, J = 5.5 Hz, 1H), 7.84-7.86 (t, J = 5.4 Hz, 1H), 8.04-8.21 (m, 5H).

^{13}C-NMR (100 MHz, DMSO) δ: 28.2, 38.7, 41.9, 42.0, 42.2, 43.1, 43.3, 43.7, 49.9, 68.7, 69.0, 78.2, 155.9, 168.7, 168.9, 169.0, 169.3, 169.4, 169.8.

HRMS: HR-MS (ESI) Calcd for $C_{21}H_{36}N_{10}O_9$ $[M+H]^+$ 573.2739, found 573.2749.

Cyanocobalamin triazole

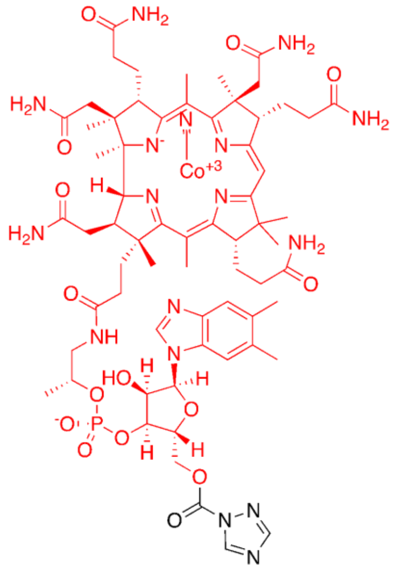

Cyanocobalamin triazole was prepared as previously described in literature.[9] A round bottom flask was dried in an oven and cooled in a dessicator. Cyanocobalamin (150 mg, 0.11 mmol) was dissolved in anhydrous DMSO (3 mL) with stirring. 1,1′-Carbonyl-di-(1,2,4-triazole) (90 mg, 0.55 mmol) was added to the solution in one portion. Note: CDT is an extremely hygroscopic reagent and decomposes rapidly to triazole upon exposure to moisture. Fresh CDT was used to avoid low conversion. The reaction was stirred at room temperature for 1h under Ar and protected from light.

The product was precipitated by dropwise addition to a gently stirring, ice cold solution of 500 mL 1:1 Et_2O:$CHCl_3$ and allowed to stand for 5 min on ice. The precipitate was collected by centrifugation (3000 *x* g, 3 min, 4°C). Residual solvent was removed on hi-vac for 15 minutes.

Cyanocobalamin triazole was isolated as a red solid (931 mg, 64%) and used crude in the next step.

General Procedure for Coupling Polyglycine Linker to Cyanocobalamin

This general procedure was used to couple a polyglycine linker to cyanocobalamin triazole.

Boc-protected polyglycine linker (0.11 mmol) was placed in a polypropylene falcon tube and deprotected with DCM (5 mL), TFA (1 mL) and water (0.2 mL) at 0°C for 1h and then room temperature for 30 min. The deprotection cocktail was removed by evaporating with a stream of air. The residue was dissolved in CH_3CN (5 mL) and water (10 mL) and lyophilized. The crude, white solid was used without further purification.

The TFA•amine salt of the polyglycine linker (0.11 mmol) was dissolved in DMSO (1.5 mL) and iPr_2NEt (0.22 mmol). Freshly prepared cyanocobalamin triazole (0.055 mmol) was added and the reaction was stirred at room temperature overnight, under Ar and protected from light.

The reaction was precipitated by dropwise addition into a stirred, ice-cold solution of 1:1 CH_3Cl:Et_2O (100 mL). The precipitate was allowed to stand for 5 minutes on ice and then collected by centrifugation (3000 *x* g, 3 min, 4°C). The pellet was rinsed twice with ice-cold Et_2O (40 mL). Residual solvent was removed on the hi-vac (protected from light) and the crude red solid was purified by reverse-phase HPLC.

Supporting Compound (5)

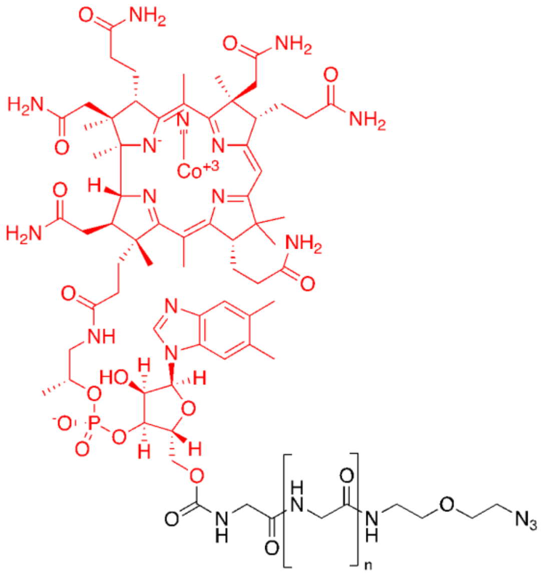

5 n = 2

The crude red solid was purified by reverse phase HPLC (gradient 0 – 80% MeOH in water containing 0.1% TFA) to afford (**5**) (38 mg, 23% yield) after lyophilization as a red solid;

Purity of the final product was established by reverse phase HPLC (gradient 0 – 100% MeOH in water containing 0.1% TFA over 30 minutes); t_R [**5**] = 15.05 min (96.8% pure).

HR-MS (ESI) Calcd for $C_{74}H_{105}CoN_{21}O_{19}P$ $[M+2H]^{2+}$ 841.8552, found 841.8552.

Supporting Compound (6)

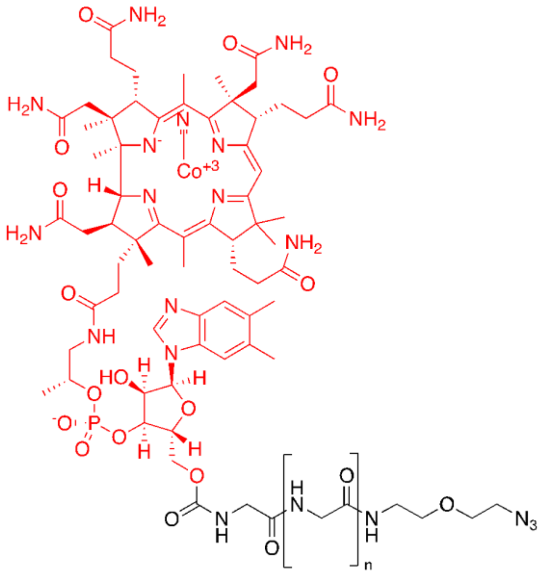

6 n = 3

The crude red solid was purified by reverse phase HPLC (gradient 0 – 80% MeOH in water containing 0.1% TFA) to afford (**6**) (34 mg, 35% yield) after lyophilization as a red solid;

Purity of the final product was established by reverse phase HPLC (gradient 0 – 100% MeOH in water containing 0.1% TFA over 30 minutes); t_R [**6**] = 15.12 min (94.1% pure).

HR-MS (ESI) Calcd for $C_{76}H_{108}CoN_{22}O_{20}P$ $[M+2H]^{2+}$ 870.3659, found 870.3689.

Supporting Compound (7)

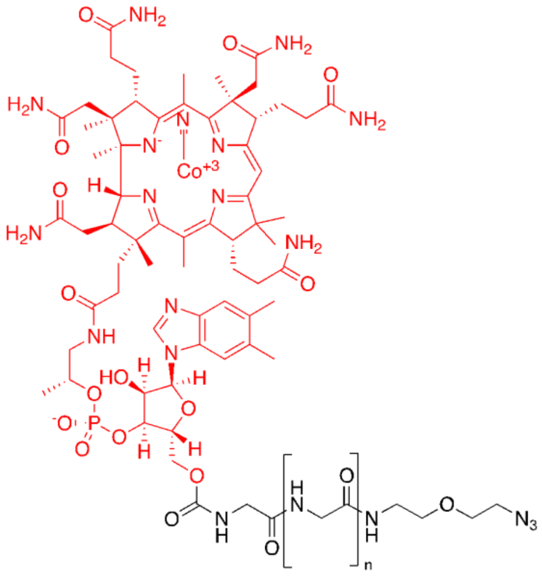

7 n = 4

The crude red solid was purified by reverse phase HPLC (gradient 0 – 80% MeOH in water containing 0.1% TFA) to afford (**7**) (44 mg, 30% yield) after lyophilization as a red solid;

Purity of the final product was established by reverse phase HPLC (gradient 0 – 100% MeOH in water containing 0.1% TFA over 30 minutes); t_R [**7**] = 14.94 min (98.7% pure).

HR-MS (ESI) Calcd for $C_{78}H_{111}CoN_{23}O_{21}P$ $[M+2H]^{2+}$ 898.8775, found 898.8764.

Supporting Compound (8)

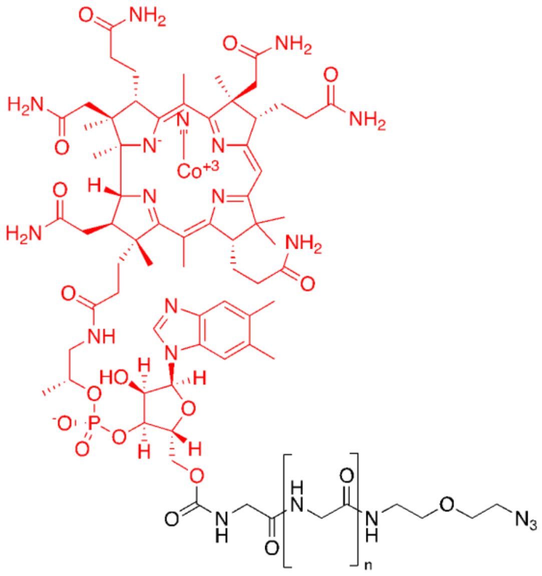

8 n = 5

The crude red solid was purified by reverse phase HPLC (gradient 0 – 80% MeOH in water containing 0.1% TFA) to afford (**8**) (40 mg, 39% yield) after lyophilization as a red solid.

Purity of the final product was established by reverse phase HPLC (gradient 0 – 100% MeOH in water containing 0.1% TFA over 30 minutes); t_R [**8**] = 14.97 min (97.4% pure).

HR-MS (ESI) Calcd for $C_{80}H_{114}CoN_{24}O_{22}P$ $[M+H]^+$ 1853.7687, found 1853.7656.

Riboglow Probes Synthesized in this Study

Cbl-5xPeg-ATTO590 (5xPeg) and Cbl-4xGly1-ATTO590 (4xGly1) were previously described in the literature.[1]

General Procedure for Click Coupling ATTO 590 Alkyne

This general procedure was used to couple ATTO 590 alkyne to each cyanocobalamin functionalized with a polyglycine azide linker: Cbl-NxGly-N_3.

TBTA (3.6 mg, 0.0068 mmol) and Cu(I) iodide (1.3 mg, 0.0068 mmol) were combined in DMF (0.28 mL) and stirred at room temperature for 30 minutes.

The functionalized cyanocobalamin (Cbl-NxGly-N_3, 0.0018 mmol) was placed in a 1.5 mL Eppendorf tube with MS grade water (130 µL) and a small magnetic stir bar. ATTO590 alkyne (0.0014 mmol) was added as a solution in DMF (125 µL). The click reaction was initiated by addition of the TBTA/Cu(I) iodide solution. The reaction was stirred for 18 hours at room temperature, protected from light.

The reaction was terminated by dropwise addition to ice-cold Et_2O (4 mL). The reaction Eppendorf was rinsed with 50 µL of ice cold MeOH, which was also added to the Et_2O. The solution was vortexed and centrifuged (3000 *x* g, 3 min, 4°C). The top Et_2O was discarded. The bottom layer was then added to an ice-cold solution of 1:1 $CHCl_3$:Et_2O, vortexed and centrifuged (3000 *x* g, 3 min, 4°C). The intensely colored top layer, containing the product, was removed by syringe to a small scintillation vial. The solvent was removed in vacuo, protected from light and then purified by reverse-phase HPLC.

Cbl-3xGly-ATTO590 (3xGly)

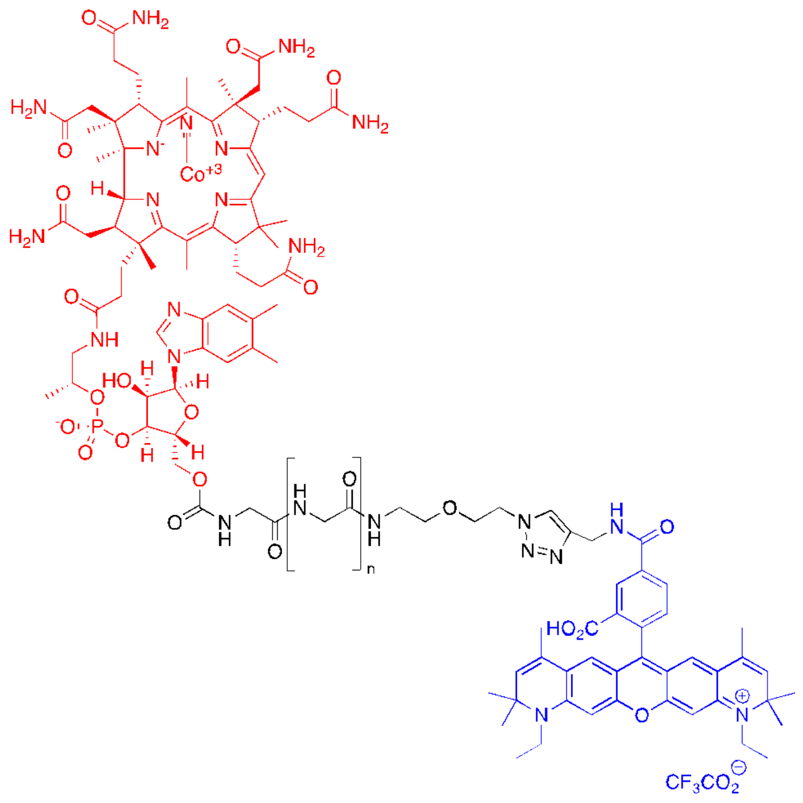

3xGly n = 2

The crude red solid was purified by reverse phase HPLC (gradient 0 – 80% MeOH in water containing 0.1% TFA) to afford (**3xGly**) (1.7 mg, 51% yield) after lyophilization as a blue solid;

Purity of the final product was established by reverse phase HPLC (gradient 0 – 100% MeOH in water containing 0.1% TFA over 30 minutes); t_R [**3xGly**] = 24.07 min (96.7% pure).

Identity of the final product was established by HR-MS (ESI):
Calcd for $C_{114}H_{147}CoN_{24}O_{23}P^{+}$ $[M+H]^{2+}$ 1155.5109, found 1155.5125.

Cbl-4xGly2-ATTO590 (4xGly2)

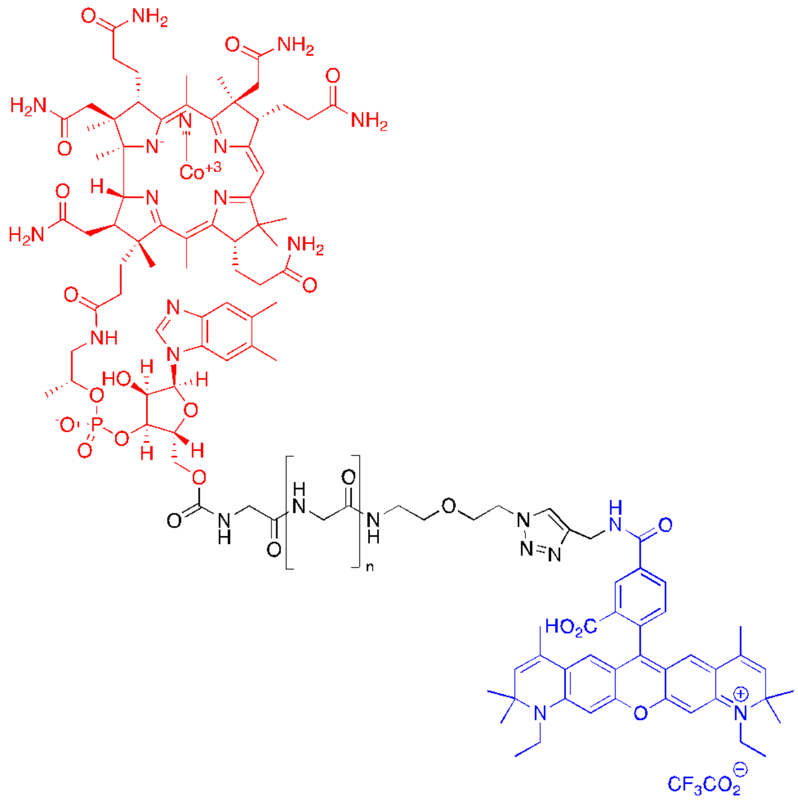

4xGly n = 3

The crude red solid was purified by reverse phase HPLC (gradient 0 – 80% MeOH in water containing 0.1% TFA) to afford (**4xGly2**) (1.1 mg, 33% yield) after lyophilization as a blue solid.

Purity of the final product was established by reverse phase HPLC (gradient 0 – 100% MeOH in water containing 0.1% TFA over 30 minutes); t_R [**4xGly2**] = 23.99 min (95.8% pure).

Identity of the final product was established by HR-MS (ESI):
Calcd for $C_{116}H_{150}CoN_{25}O_{24}P^+$ $[M+H]^{2+}$ 1184.0211, found 1184.0199.

Cbl-5xGly-ATTO590 (5xGly)

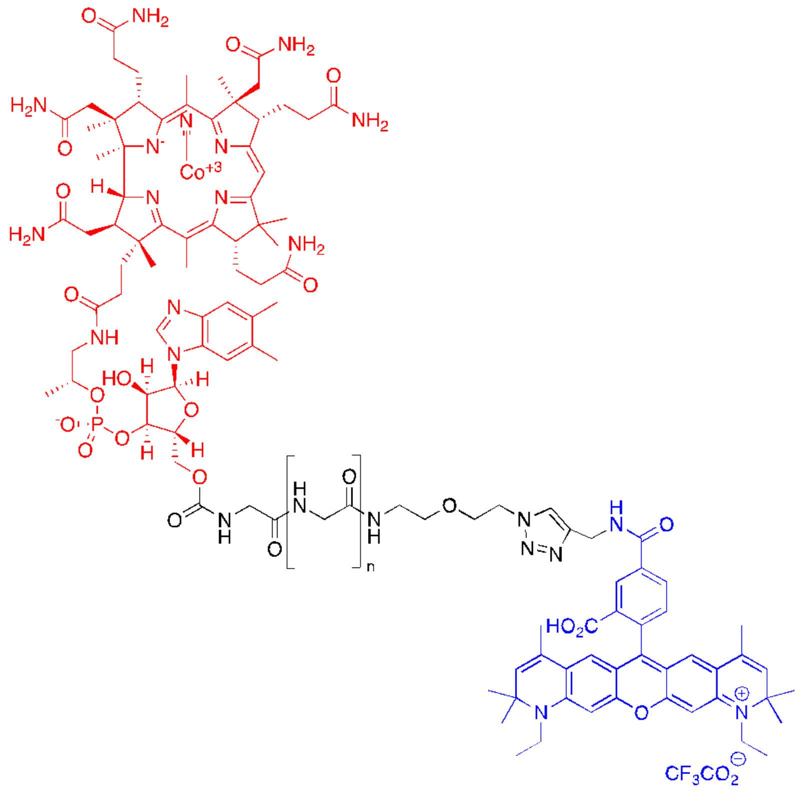

5xGly n = 4

The crude red solid was purified by reverse phase HPLC (gradient 0 – 80% MeOH in water containing 0.1% TFA) to afford (**5xGly**) (1.4 mg, 42% yield) after lyophilization as a blue solid.

Purity of the final product was established by reverse phase HPLC (gradient 0 – 100% MeOH in water containing 0.1% TFA over 30 minutes); t_R [**5xGly**] = 23.92 min (96.3% pure).

Identity of the final product was established by HR-MS (ESI):
Calcd for $C_{118}H_{152}CoN_{26}O_{25}P^+$ $[M+H]^{2+}$ 1212.0279, found 212.0261.

Cbl-6xGly-ATTO590 (6xGly)

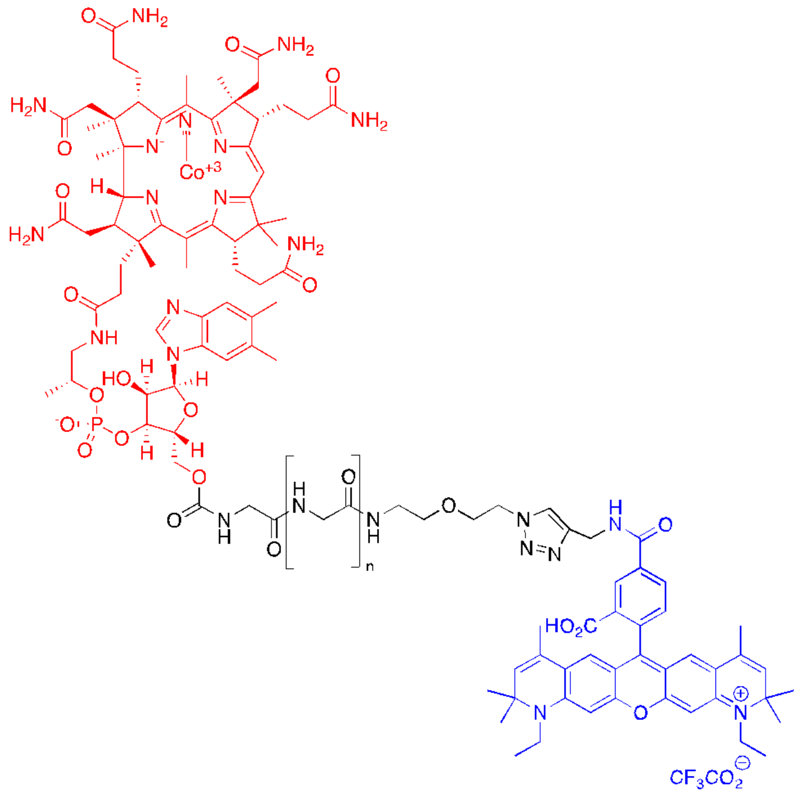

6xGly n = 5

The crude red solid was purified by reverse phase HPLC (gradient 0 – 80% MeOH in water containing 0.1% TFA) to afford (**6xGly**) (1.3 mg, 39% yield) after lyophilization as a blue solid.

Purity of the final product was established by reverse phase HPLC (gradient 0 – 100% MeOH in water containing 0.1% TFA over 30 minutes); t_R [**6xGly**] = 23.84 min (96.4% pure).

Identity of the final product was established by HR-MS (ESI):
Calcd for $C_{120}H_{156}CoN_{27}O_{26}P^{+}$ $[M+Na]^{2+}$ 1252.0336, found 1252.0329.

Supplementary References

(1) Braselmann, E.; Wierzba, A. J.; Polaski, J. T.; Chromiński, M.; Holmes, Z. E.; Hung, S.-T.; Batan, D.; Wheeler, J. R.; Parker, R.; Jimenez, R.; Gryko, D.; Batey, R. T.; Palmer, A. E. A Multicolor Riboswitch-Based Platform for Imaging of RNA in Live Mammalian Cells. *Nat. Chem. Biol.* **2018**, *14* (10), 964–971. https://doi.org/10.1038/s41589-018-0103-7.

(2) Stickelman, Z.; Sarfraz, N.; Rice, M. K.; Lambeck, B. J.; Milkovich, S.; Braselmann, E. Expanding the Riboglow-FLIM Toolbox with Different Fluorescence Lifetime-Producing RNA Tags. *Biochemistry* **2025**, acs.biochem.4c00567. https://doi.org/10.1021/acs.biochem.4c00567.

(3) Mark, J. E.; Flory, P. J. The Configuration of the Polyoxyethylene Chain. *J. Am. Chem. Soc.* **1965**, *87* (7), 1415–1423. https://doi.org/10.1021/ja01085a001.

(4) Everaers, R.; Grosberg, A. Y.; Rubinstein, M.; Rosa, A. Flory Theory of Randomly Branched Polymers. *Soft Matter* **2017**, *13* (6), 1223–1234. https://doi.org/10.1039/C6SM02756C.

(5) Vitalis, A.; Wang, X.; Pappu, R. V. Atomistic Simulations of the Effects of Polyglutamine Chain Length and Solvent Quality on Conformational Equilibria and Spontaneous Homodimerization. *J. Mol. Biol.* **2008**, *384* (1), 279–297. https://doi.org/10.1016/j.jmb.2008.09.026.

(6) Lennon, S. R.; Wierzba, A. J.; Siwik, S. H.; Gryko, D.; Palmer, A. E.; Batey, R. T. Targeting Riboswitches with Beta-Axial-Substituted Cobalamins. *ACS Chem. Biol.* **2023**, *18* (5), 1136–1147. https://doi.org/10.1021/acschembio.2c00939.

(7) Sarfraz, N.; Shafik, L. K.; Stickelman, Z. R.; Shankar, U.; Moscoso, E.; Braselmann, E. Evaluating Riboglow-FLIM Probes for RNA Sensing. *RSC Chem. Biol.* **2024**, *5* (2), 109–116. https://doi.org/10.1039/D3CB00197K.

(8) O'Leary, M. K.; Chen, S. S.; Westblade, L. F.; Alabi, C. A. Design of a PEGylated Antimicrobial Prodrug with Species-Specific Activation. *Biomacromolecules* **2021**, *22* (2), 984–992. https://doi.org/10.1021/acs.biomac.0c01695.

(9) Fazen, C. H.; Valentin, D.; Fairchild, T. J.; Doyle, R. P. Oral Delivery of the Appetite Suppressing Peptide hPYY(3–36) through the Vitamin B_{12} Uptake Pathway. *J. Med. Chem.* **2011**, *54* (24), 8707–8711. https://doi.org/10.1021/jm2012547.

HPLC Chromatograms

Supporting Compound (5)

SHIMADZU LabSolutions **Analysis Report**

<Sample Information>

Sample Name	: SI_HPLC 1		
Sample ID	: SI_HPLC 1		
Data Filename	: SI_HPLC 1.lcd		
Method Filename	: AVD 0-100 MeOH wTFA 30min.lcm		
Batch Filename	: SI Batch 1.lcb		
Vial #	: 1-32	Sample Type	: Unknown
Injection Volume	: 25 uL		
Date Acquired	: 5/14/2025 4:40:55 PM	Acquired by	: System Administrator
Date Processed	: 5/14/2025 5:21:02 PM	Processed by	: System Administrator

<Chromatogram>

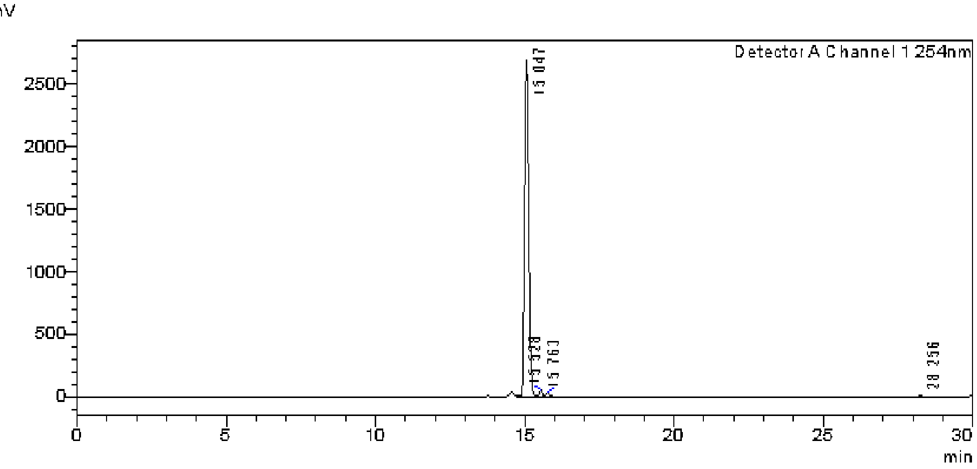

<Peak Table>

Detector A Channel 1 254nm

Peak#	Ret. Time	Area	Height	Area%
1	15.047	25943213	2692385	96.849
2	15.528	446804	60292	1.668
3	15.763	308281	33776	1.151
4	28.256	88977	16384	0.332
Total		26787275	2802837	100.000

Supporting Compound (6)

SHIMADZU LabSolutions

Analysis Report

<Sample Information>

Sample Name	: SI_HPLC 3		
Sample ID	: SI_HPLC 3		
Data Filename	: SI_HPLC 3.lcd		
Method Filename	: AVD 0-100 MeOH w TFA 30min.lcm		
Batch Filename	: SI Batch 1.lcb		
Vial #	: 1-32	Sample Type	: Unknown
Injection Volume	: 75 uL		
Date Acquired	: 5/14/2025 6:02:12 PM	Acquired by	: System Administrator
Date Processed	: 5/14/2025 6:42:19 PM	Processed by	: System Administrator

<Chromatogram>

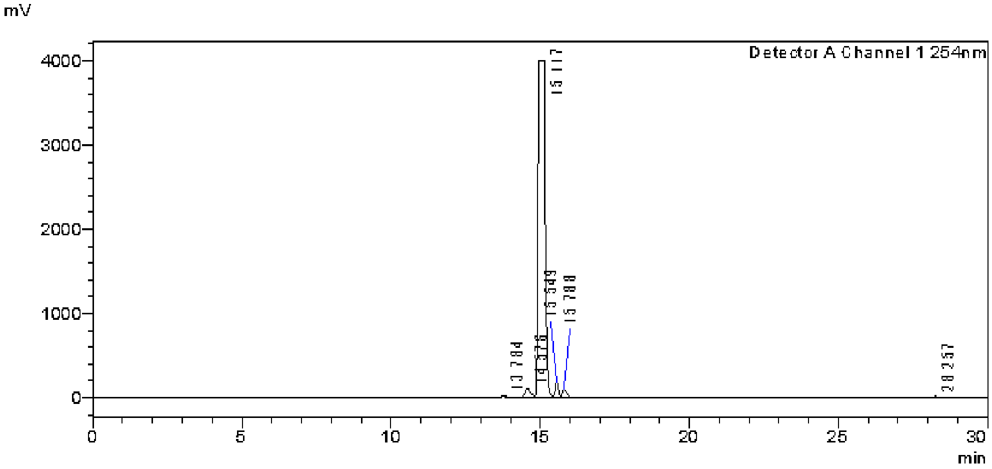

<Peak Table>

Detector A Channel 1 254nm

Peak#	Ret. Time	Area	Height	Area%
1	13.784	132926	24163	0.202
2	14.576	1332196	106373	2.020
3	15.117	62054596	3994899	94.072
4	15.549	1392523	180871	2.111
5	15.788	968275	103507	1.468
6	28.257	84509	15800	0.128
Total		65965024	4425613	100.000

Supporting Compound (7)

Analysis Report

<Sample Information>

Sample Name	: SI_HPLC 2		
Sample ID	: SI_HPLC 2		
Data Filename	: SI_HPLC 2.lcd		
Method Filename	: AVD 0-100 MeOH wTFA 30min.lcm		
Batch Filename	: SI Batch 1.lcb		
Vial #	: 1-33	Sample Type	: Unknown
Injection Volume	: 25 uL		
Date Acquired	: 5/14/2025 5:21:32 PM	Acquired by	: System Administrator
Date Processed	: 5/14/2025 6:01:39 PM	Processed by	: System Administrator

<Chromatogram>

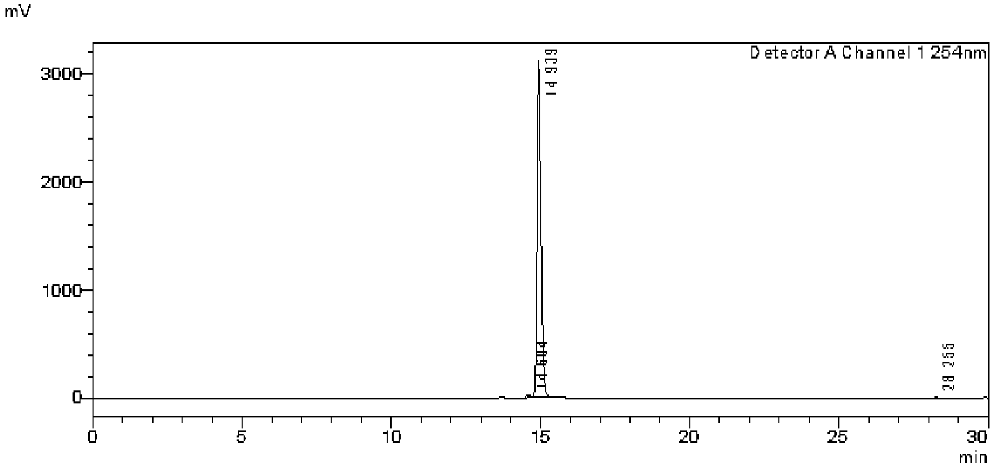

<Peak Table>

Detector A Channel 1 254nm

Peak#	Ret. Time	Area	Height	Area%
1	14.604	278022	29900	0.982
2	14.939	27953886	3104467	98.715
3	28.255	85868	16098	0.303
Total		28317775	3150466	100.000

Supporting Compound (8)

Analysis Report

<Sample Information>

Sample Name	: SI_HPLC 4		
Sample ID	: SI_HPLC 4		
Data Filename	: SI_HPLC 4.lcd		
Method Filename	: AVD 0-100 MeOH w TFA 30min.lcm		
Batch Filename	: SI Batch 1.lcb		
Vial #	: 1-33	Sample Type	: Unknown
Injection Volume	: 75 uL		
Date Acquired	: 5/14/2025 6:42:51 PM	Acquired by	: System Administrator
Date Processed	: 5/14/2025 7:22:57 PM	Processed by	: System Administrator

<Chromatogram>

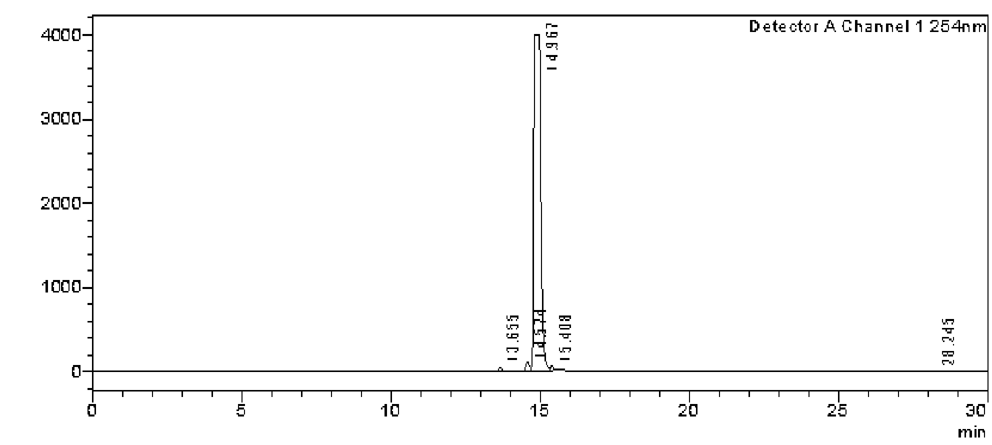

<Peak Table>

Detector A Channel 1 254nm

Peak#	Ret. Time	Area	Height	Area%
1	13.655	210229	37334	0.325
2	14.574	1017659	103339	1.574
3	14.967	62975045	3989920	97.388
4	15.408	375870	40841	0.581
5	28.245	85518	16013	0.132
Total		64664321	4187447	100.000

Cbl-3xGly-ATTO590 (3xGly)

Analysis Report

<Sample Information>

Sample Name	: SI_HPLC 7		
Sample ID	: SI_HPLC 7		
Data Filename	: SI_HPLC 7.lcd		
Method Filename	: AVD 0-100 MeOH w TFA 30min.lcm		
Batch Filename	: SI Batch 3.lcb		
Vial #	: 1-32	Sample Type	: Unknown
Injection Volume	: 60 uL		
Date Acquired	: 5/15/2025 3:01:11 PM	Acquired by	: System Administrator
Date Processed	: 5/15/2025 3:41:18 PM	Processed by	: System Administrator

<Chromatogram>

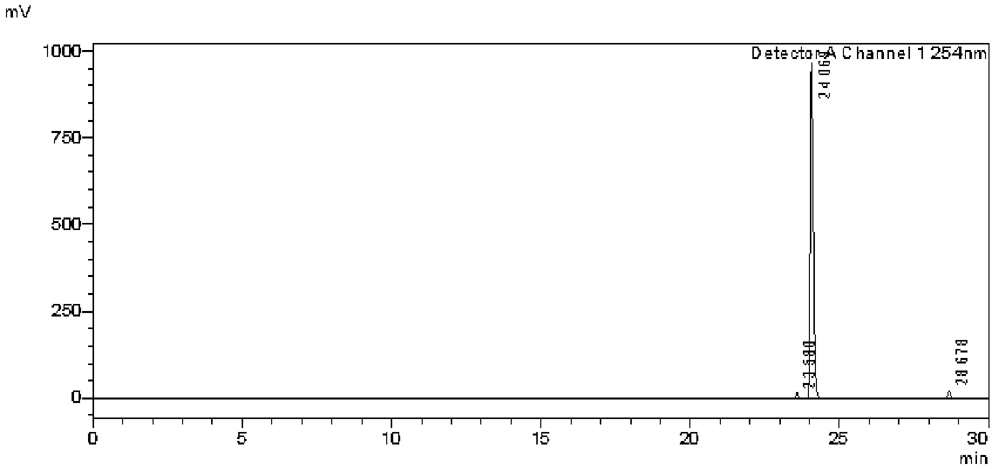

<Peak Table>

Detector A Channel 1 254nm

Peak#	Ret. Time	Area	Height	Area%
1	23.580	102458	15342	1.405
2	24.069	7054061	967931	96.758
3	28.678	133921	21290	1.837
Total		7290440	1004562	100.000

Cbl-4xGly-ATTO590 (4xGly2)

SHIMADZU LabSolutions **Analysis Report**

<Sample Information>

Sample Name	: SI_HPLC 8		
Sample ID	: SI_HPLC 8		
Data Filename	: SI_HPLC 8.lcd		
Method Filename	: AVD 0-100 MeOH wTFA 30min.lcm		
Batch Filename	: SI Batch 3.lcb		
Vial #	: 1-33	Sample Type	: Unknown
Injection Volume	: 60 uL		
Date Acquired	: 5/15/2025 3:41:49 PM	Acquired by	: System Administrator
Date Processed	: 5/15/2025 4:21:55 PM	Processed by	: System Administrator

<Chromatogram>

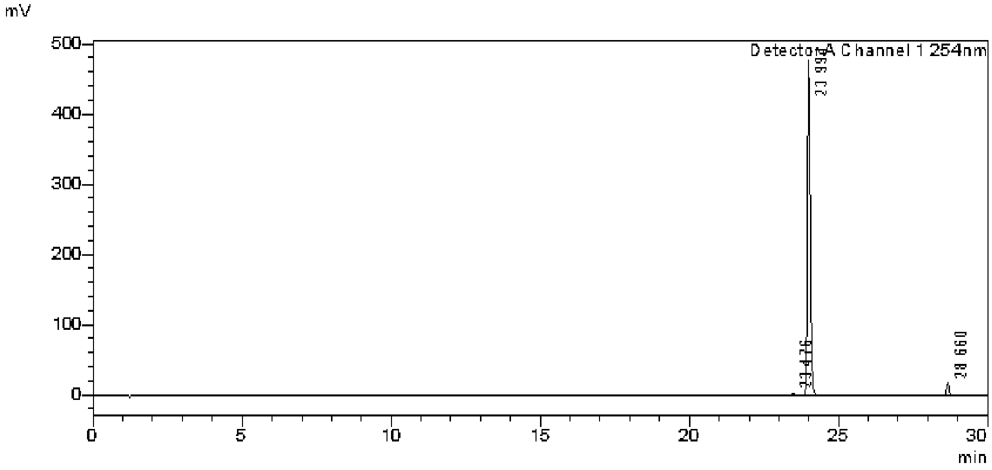

<Peak Table>

Detector A Channel 1 254nm

Peak#	Ret. Time	Area	Height	Area%
1	23.476	27101	3740	0.815
2	23.994	3187774	477941	95.830
3	28.660	111602	17705	3.355
Total		3326477	499387	100.000

Cbl-5xGly-ATTO590 (5xGly)

Analysis Report

SHIMADZU LabSolutions

<Sample Information>

Sample Name	: SI_HPLC 9		
Sample ID	: SI_HPLC 9		
Data Filename	: SI_HPLC 9.lcd		
Method Filename	: AVD 0-100 MeOH wTFA 30min.lcm		
Batch Filename	: SI Batch 3.lcb		
Vial #	: 1-34	Sample Type	: Unknown
Injection Volume	: 60 uL		
Date Acquired	: 5/15/2025 4:22:27 PM	Acquired by	: System Administrator
Date Processed	: 5/15/2025 5:02:34 PM	Processed by	: System Administrator

<Chromatogram>

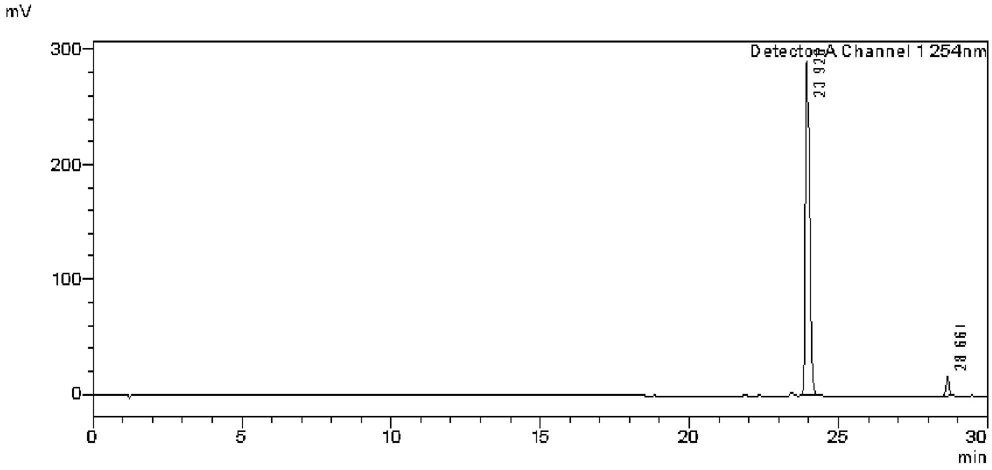

<Peak Table>

Detector A Channel 1 254nm

Peak#	Ret. Time	Area	Height	Area%
1	23.926	2931318	290956	96.322
2	28.661	111944	17740	3.678
Total		3043263	308695	100.000

Cbl-6xGly-ATTO590 (6xGly)

SHIMADZU LabSolutions

Analysis Report

<Sample Information>

Sample Name	: SI_HPLC 10		
Sample ID	: SI_HPLC 10		
Data Filename	: SI_HPLC 10.lcd		
Method Filename	: AVD 0-100 MeOH wTFA 30min.lcm		
Batch Filename	: SI Batch 3.lcb		
Vial #	: 1-35	Sample Type	: Unknown
Injection Volume	: 60 uL		
Date Acquired	: 5/15/2025 5:03:04 PM	Acquired by	: System Administrator
Date Processed	: 5/15/2025 5:43:11 PM	Processed by	: System Administrator

<Chromatogram>

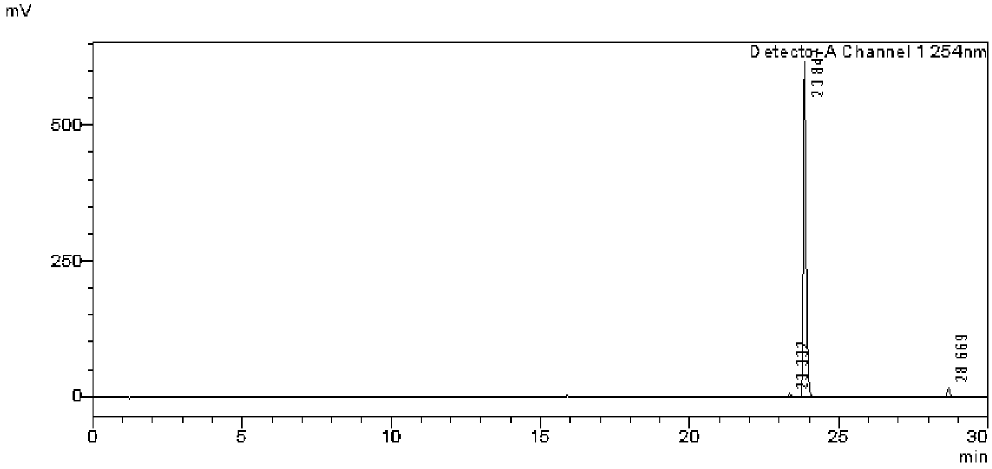

<Peak Table>

Detector A Channel 1 254nm

Peak#	Ret. Time	Area	Height	Area%
1	23.332	45610	5608	1.036
2	23.847	4243180	619782	96.412
3	28.669	112304	17828	2.552
Total		4401094	643218	100.000

^{1}H-NMR Spectra

Supporting Compound (1)

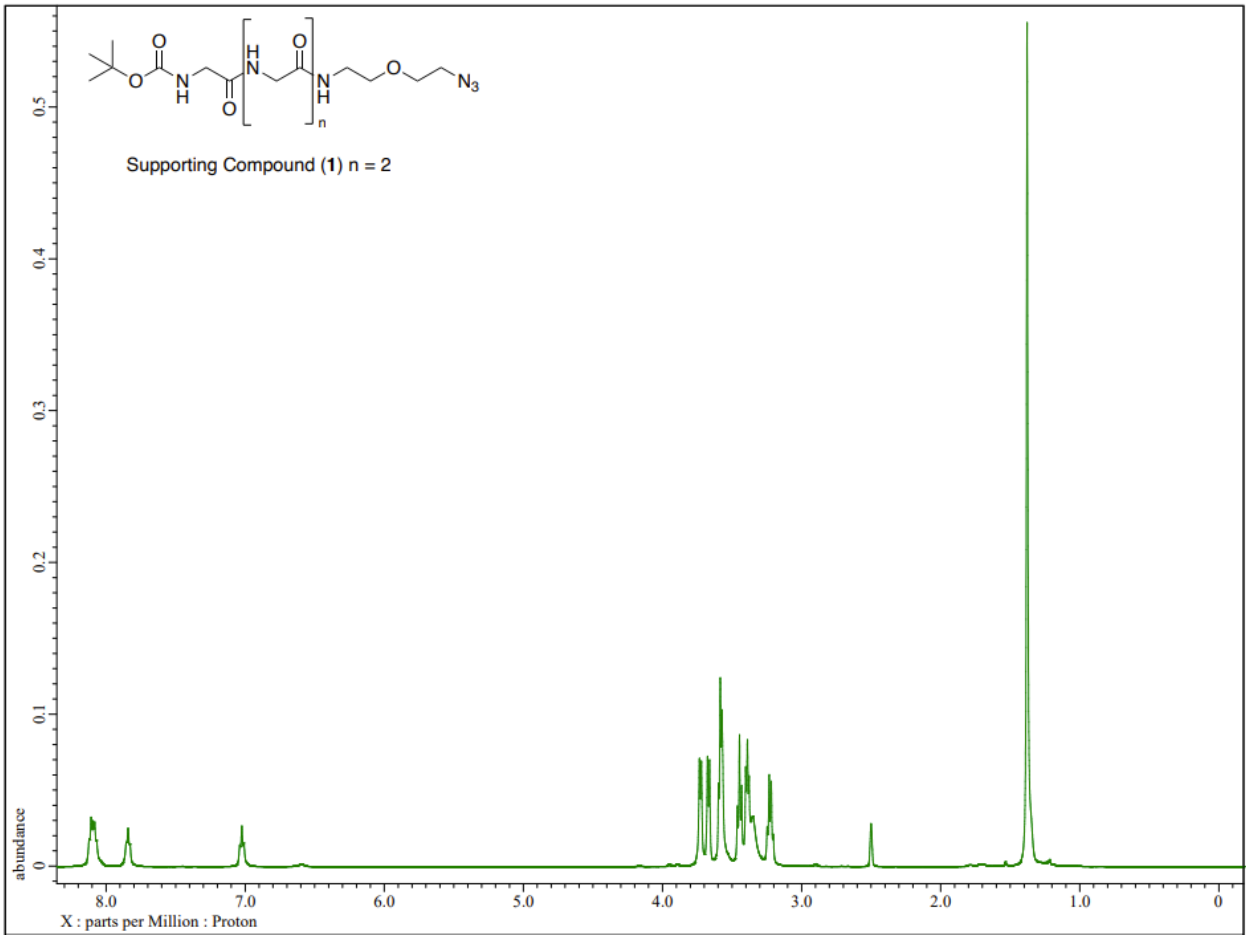

Supporting Compound (2)

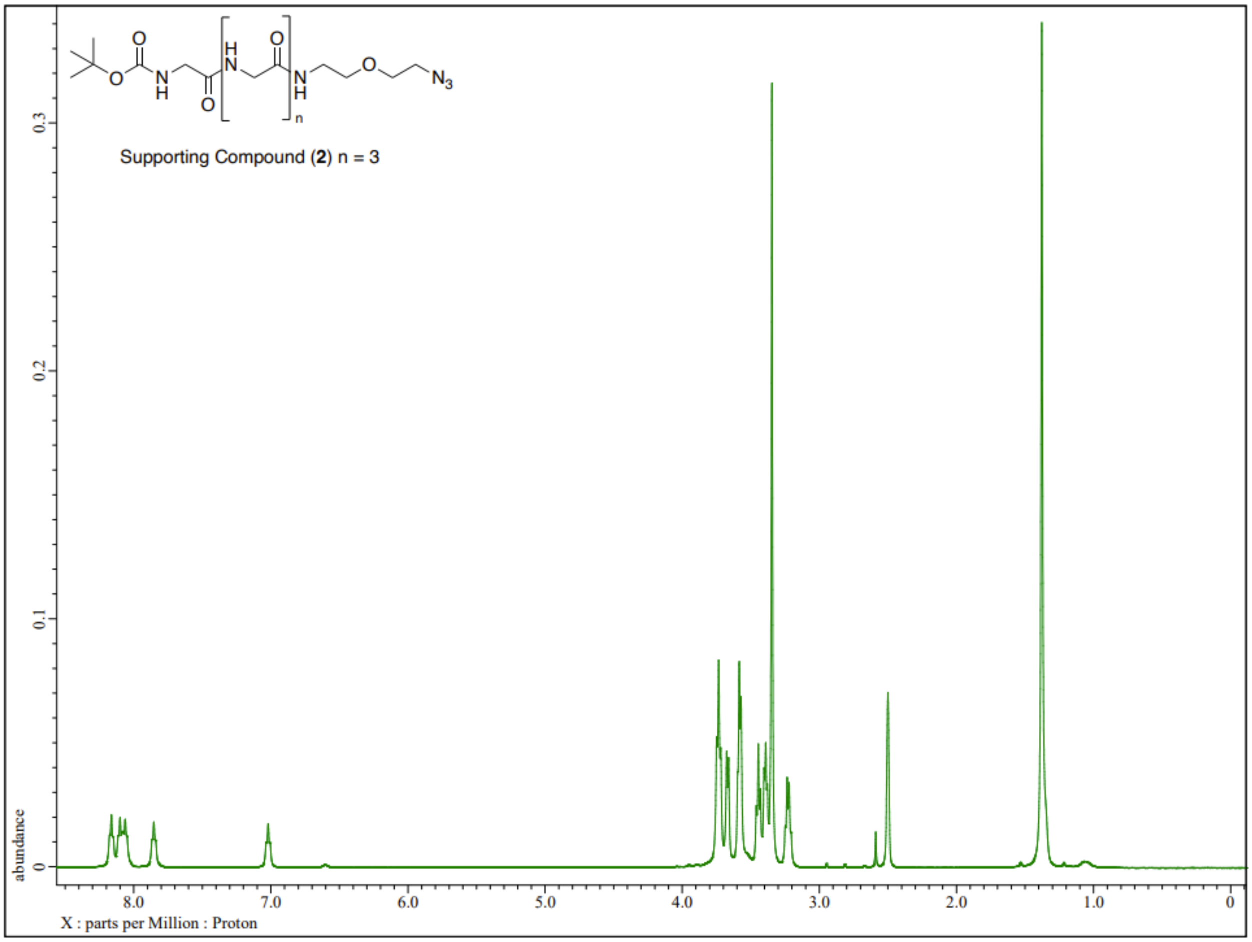

Supporting Compound (3)

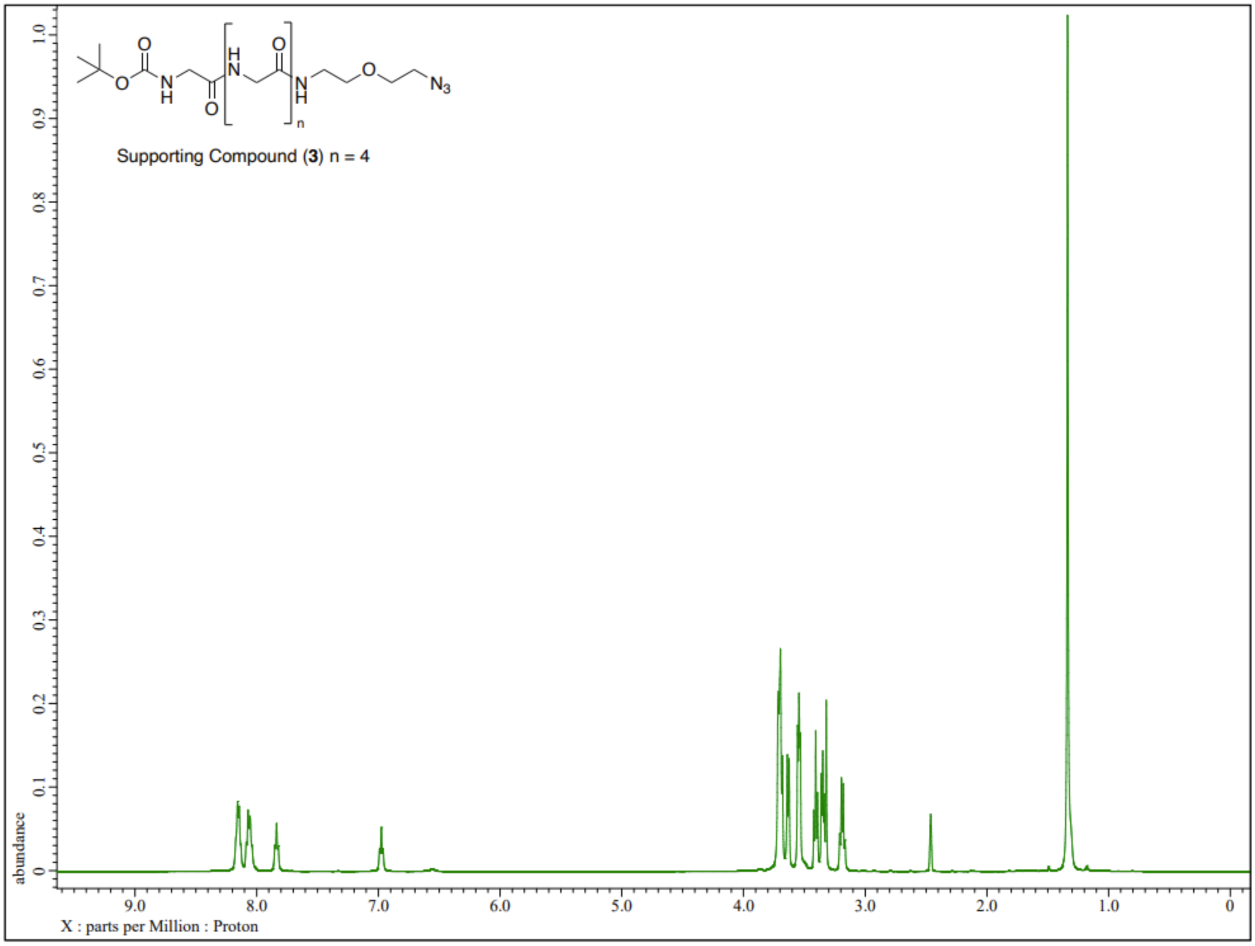

Supporting Compound (4)

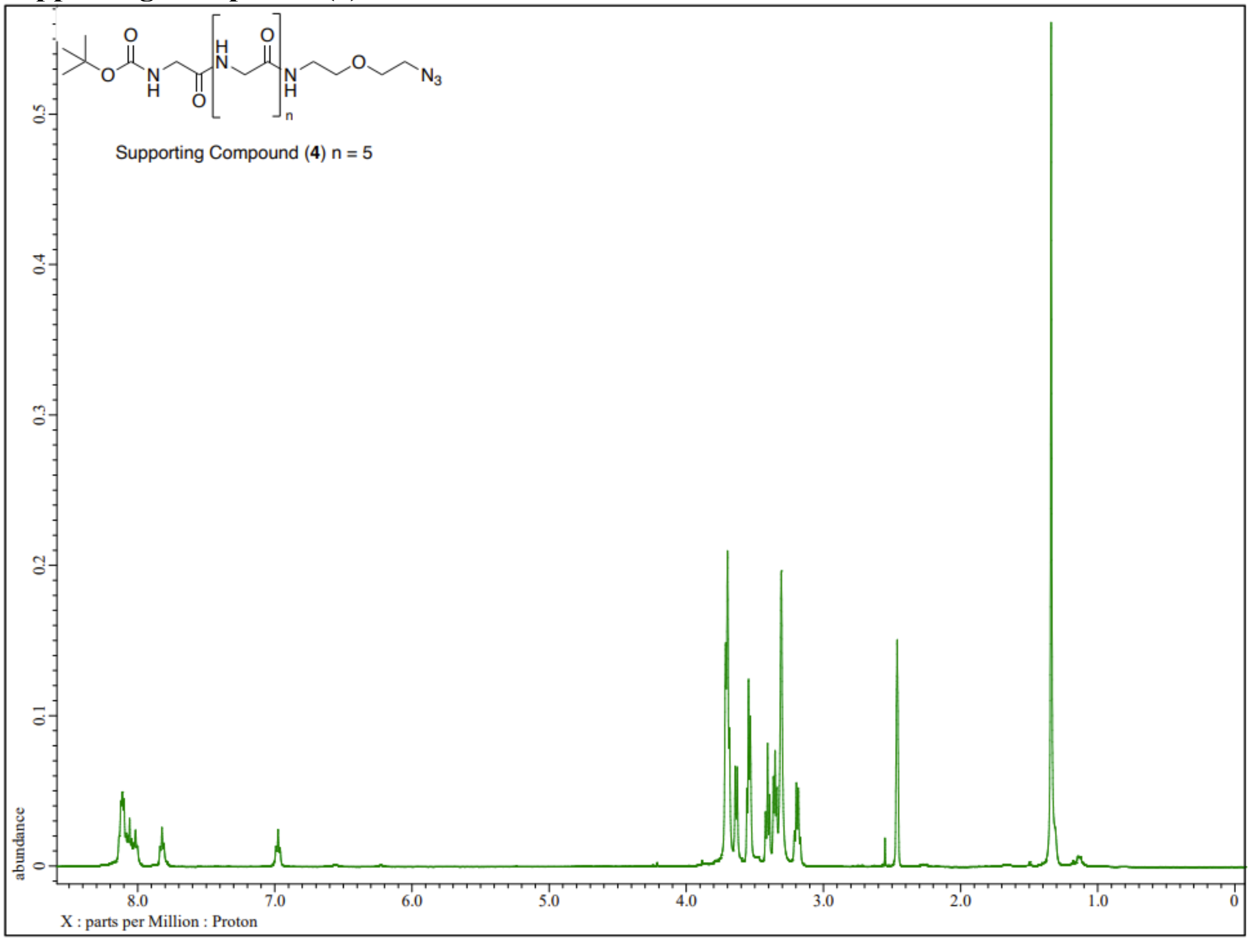

^{13}C-NMR Spectra

Supporting Compound (1)

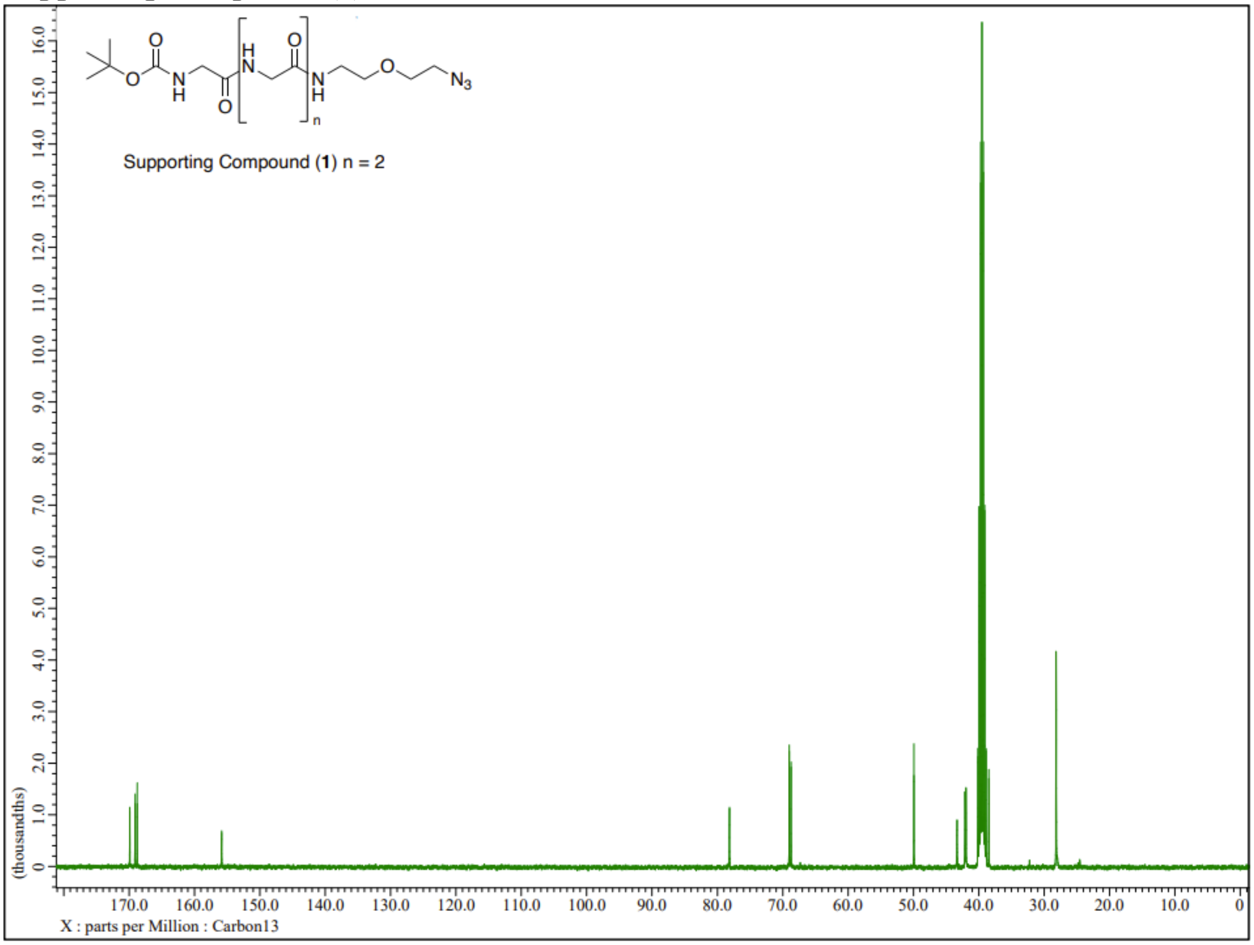

Supporting Compound (2)

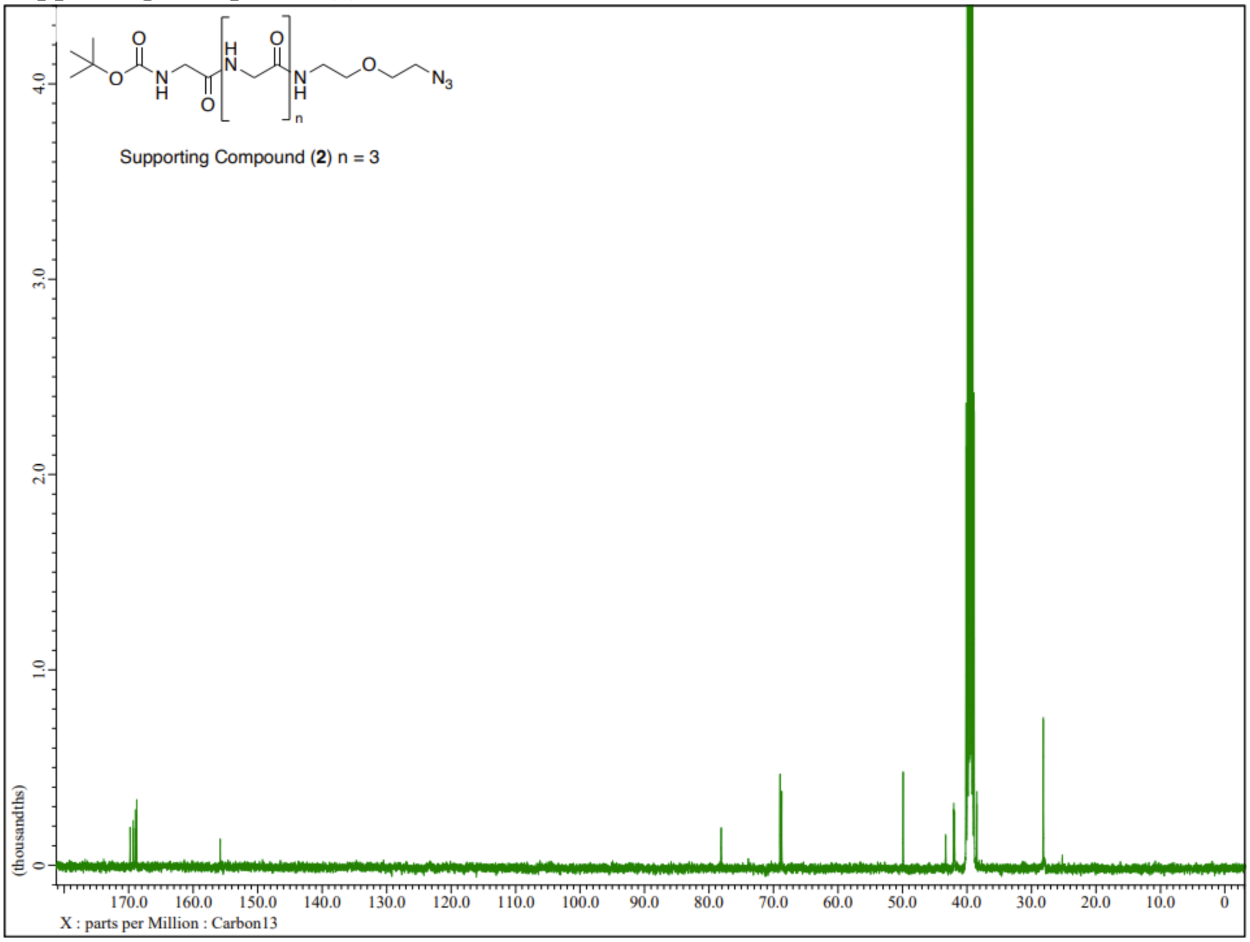

Supporting Compound (3)

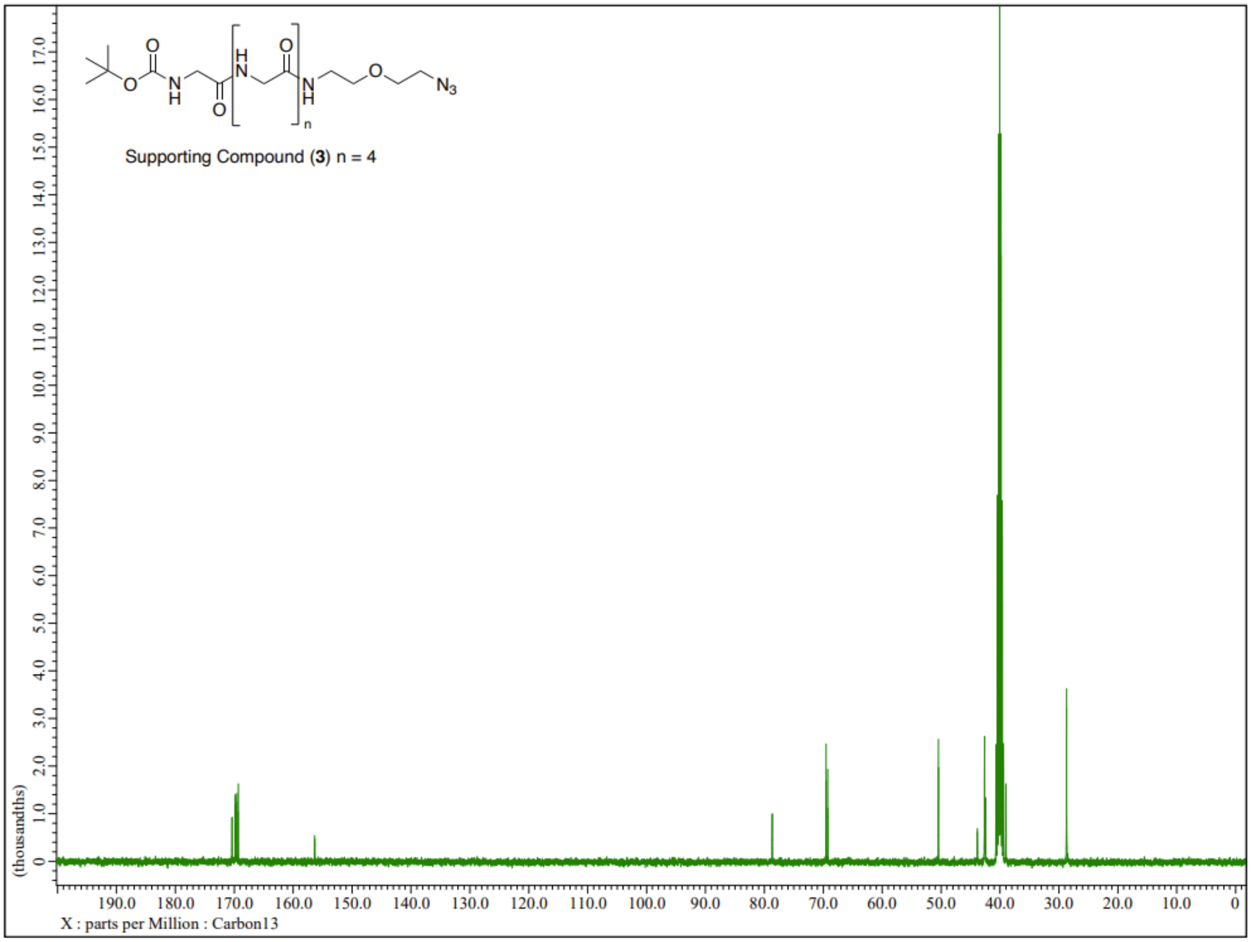

Supporting Compound (4)

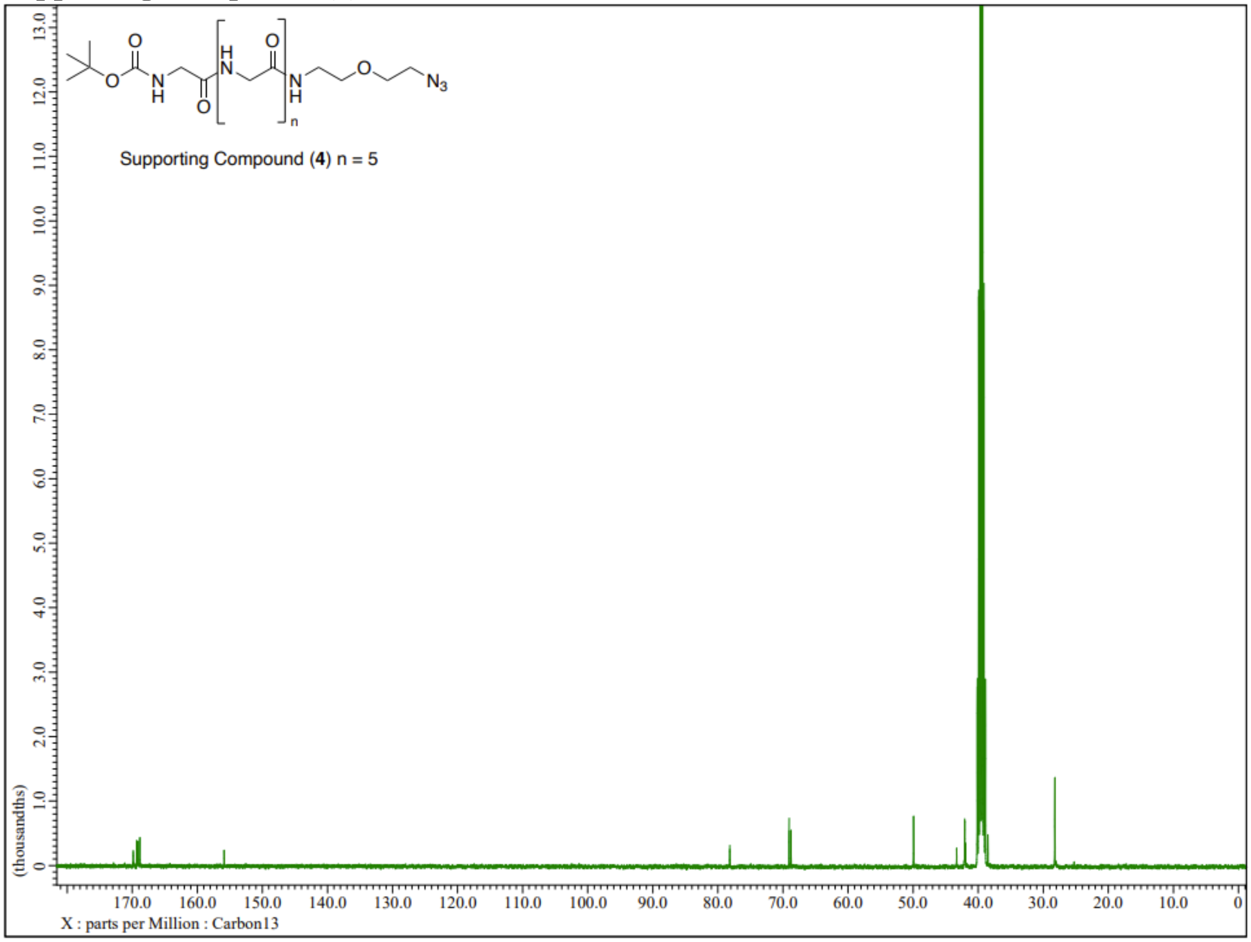

Supplement: Supplementary file 1 [file cb5c00917_si_001.pdf]
